# *Mycobacterium tuberculosis* Malate Synthase Structures with Fragments Reveal a Portal for Substrate/Product Exchange*[Fn FN2]

**DOI:** 10.1074/jbc.M116.750877

**Published:** 2016-10-13

**Authors:** Hsiao-Ling Huang, Inna V. Krieger, Maloy K. Parai, Vijay B. Gawandi, James C. Sacchettini

**Affiliations:** From the Departments of ‡ Chemistry and; § Biochemistry and Biophysics, Texas A&M University, College Station, Texas 77845

**Keywords:** crystal structure, drug design, enzyme inhibitor, enzyme mechanism, tuberculosis, malate synthase

## Abstract

Fragment screening and high throughput screening are complementary approaches that combine with structural biology to explore the binding capabilities of an active site. We have used a fragment-based approach on malate synthase (GlcB) from *Mycobacterium tuberculosis* and discovered several novel binding chemotypes. In addition, the crystal structures of GlcB in complex with these fragments indicated conformational changes in the active site that represent the enzyme conformations during catalysis. Additional structures of the complex with malate and of the apo form of GlcB supported that hypothesis. Comparative analysis of GlcB structures in complex with 18 fragments allowed us to characterize the preferred chemotypes and their binding modes. The fragment structures showed a hydrogen bond to the backbone carbonyl of Met-631. We successfully incorporated an indole group from a fragment into an existing phenyl-diketo acid series. The resulting indole-containing inhibitor was 100-fold more potent than the parent phenyl-diketo acid with an IC_50_ value of 20 nm.

## Introduction

Advances in high throughput screening and structural biology have made fragment-based methods an integral part of many structure-guided drug discovery programs (1). These methods take advantage of the structure of drug targets bound with small molecules to define new groups that interact with the protein. Typically, fragments bind with relatively weak affinity (*K_d_* > 0.1 mm) (2). Although fragment-based screening has contributed to lead discovery of numerous novel inhibitors (3–7), linking fragments into potent inhibitors has proved challenging as fragments do not always retain their binding modes during the deconstruction-reconstruction process (8, 9). Nevertheless, fragments provide valuable information about the bound states of small molecules for medicinal chemistry and chemi-informatics with two approved drugs and more than 30 in clinical trials (for recent advances, see the review by Erlanson *et al.* (10)).

We previously used structure-guided inhibitor design to develop a series of potent phenyl-diketo acid (PDKA)^3^ inhibitors that target *M. tuberculosis* malate synthase (GlcB), part of the glyoxylate shunt. This resulted in a lead molecule with efficacy in a mouse model of tuberculosis infection (11). The importance of the glyoxylate shunt was first demonstrated for chronic stages of infection and then for establishing infection through isocitrate lyase (ICL) knock-out studies (12, 13). This work agreed with the earlier observations of Segal and Bloch (14) that a metabolic shift occurs, changing the preferred carbon source from carbohydrates to fatty acids in *M. tuberculosis* recovered from infected lungs. A number of studies have confirmed the importance of the ability of *M. tuberculosis* to efficiently co-catabolize fatty acids with carbohydrates both for establishing infection and for persistence in macrophages (15–18). GlcB knockdown and knock-out studies show that loss of malate synthase function results in clearing of *M. tuberculosis* in a mouse model of infection.^4^ GlcB generates malate and coenzyme A (CoA) from glyoxylate and acetyl coenzyme A (AcCoA) following the conversion of isocitrate to succinate and glyoxylate by ICL. Because of the essentiality of the glyoxylate shunt in infection and the absence of it in humans (19), both ICL and GlcB are attractive targets for drug discovery.

Crystal structures of both glyoxylate-bound and product-bound GlcB show that there is no change between these two states either in the overall protein structure or in the active site (20). Despite a well-defined active site, multiple efforts in virtual screening have failed to produce hits with reasonable activity (21).^5^ All known structures of GlcB to date have been obtained in complex with high affinity ligands (substrate, products, and PDKA inhibitors). This tends to stabilize a preferred protein conformation, limiting any computational docking or design to one defined state.

To advance our lead development efforts on *M. tuberculosis* GlcB, we used a fragment-based approach resulting in the discovery of diverse binding chemotypes; we incorporated one novel interaction observed between the indole-containing fragments and GlcB into the existing PDKA series of inhibitors. The resulting molecule was 100 times more potent than the parent PDKA and was shown to make the predicted interactions as well as induce the same movement in the active site as the parent fragment.

Unexpectedly, the structures of malate synthase with this group of fragments captured previously unobserved conformations of the enzyme. These structures revealed a second portal to the buried active site that we hypothesized is used for substrate/product exchange. It prompted us to solve additional structures of the enzyme at various stages of product formation and dissociation as well as an apo enzyme structure. As a result, we propose a mechanism driving substrate/product exchange during catalysis.

## Results

### 

#### 

##### Binding Assay and Summary of Fragment Screening

Differential scanning fluorimetry (DSF) (22) using a conventional real time PCR instrument and the fluorescent dye SYPRO Orange was used to screen 1580 fragments for binding to GlcB. The library consisted of 757 compounds from the Maybridge MB RO3 fragment library extended by Chris Abell (Cambridge, UK) with a number of fragments with under-represented bioactive scaffolds and ring systems. It also contained 823 compounds from the Enamine Building Blocks collection picked based on the lead-like set from the ZINC 8 database (36). The fragments were filtered by molecular weight (<300), Tanimoto similarity (23), and intrinsic reactivity. The final set of 1580 fragments had a Tanimoto similarity threshold of 0.46. Binding was determined by analyzing the shift in the melting temperature (*T_m_*) of GlcB after incubation with fragments. Free GlcB unfolds at 50–55 °C, and the addition of substrates acetyl-CoA and glyoxylate resulted in Δ*T_m_* values of 2.5 and 4 °C, respectively. A known inhibitor, 2-bromo-PDKA (11), which has a *K_d_* of 0.9 μm, gave a Δ*T_m_* of about 14 °C.

The Δ*T_m_* from the screen ranged between −3.2 and 8.6 °C. 65 fragments were selected as “hits” based on a Δ*T_m_* of greater than 3 °C (Table 1 shows hits that resulted in GlcB complex crystal structures, and the rest of the hits are listed in supplemental Table S1). Examination of the chemical structures of the hits showed that all had at least one substituted aromatic ring, which included five-membered rings such as pyrrole, thiophene, and furan; six-membered rings such as benzene, pyridine, and pyrimidine; and fused ring systems such as naphthalene, indole, and thienopyrrole. In fact, more than half were substituted diaryl or triaryl compounds. The rings contained a wide variety of substituents including halogens, hydroxyls, alkyl groups, nitrile groups, acyl groups, and amine groups. Compared with the library, the hits were enriched for compounds with highly conjugated ring systems appended with carboxylate substitutions.

**TABLE 1:**
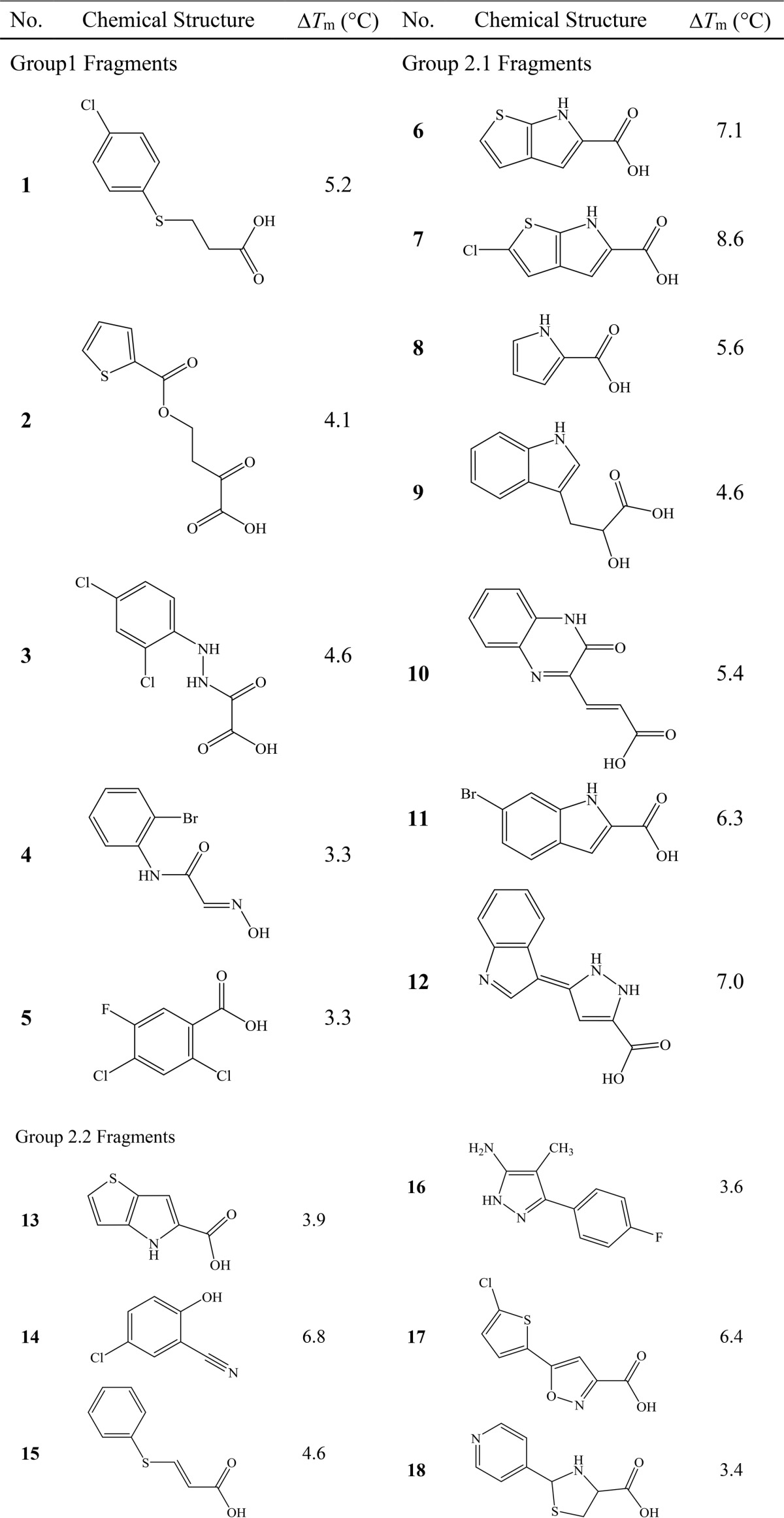
**Chemical structure and Δ*T_m_* for fragments with GlcB-complexed crystal structures**

##### Overall GlcB-Fragment Complex Crystal Structures and Fragment-induced Conformations of the Enzyme

**FIGURE 1.**
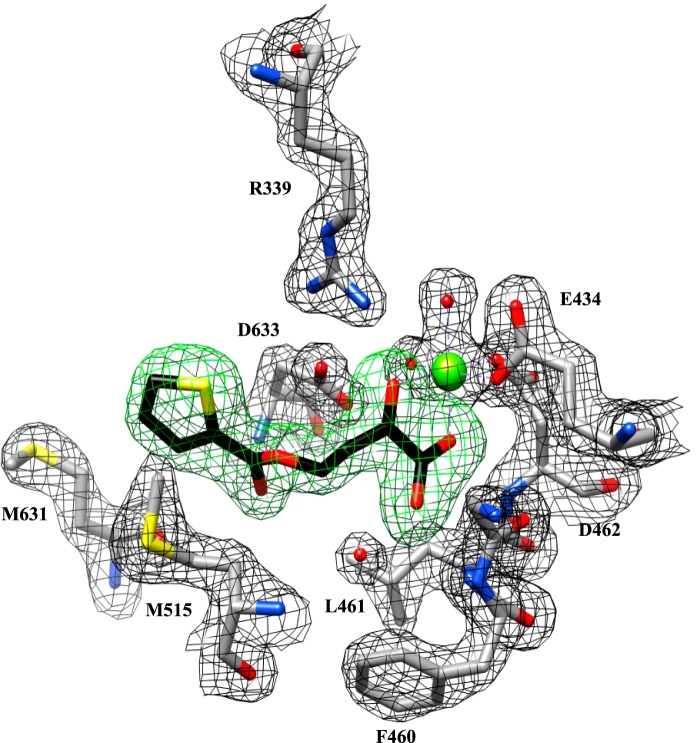
The electron density of the 2*F_o_* − *F_c_* map at 1.5 σ of GlcB in complex with fragment **2** is in *black*. The unbiased omit *F_o_* − *F_c_* difference electron density map contoured at 3.5 σ for the fragment is in *green*. Carbon atoms for the fragment are in *black*. For the protein, carbon atoms are in *gray*, and non-carbon atoms are colored as follows: magnesium, *chartreuse*; oxygen, *red*; nitrogen, *blue*; sulfur, *yellow*. Images are rendered in Chimera.

**FIGURE 2.**
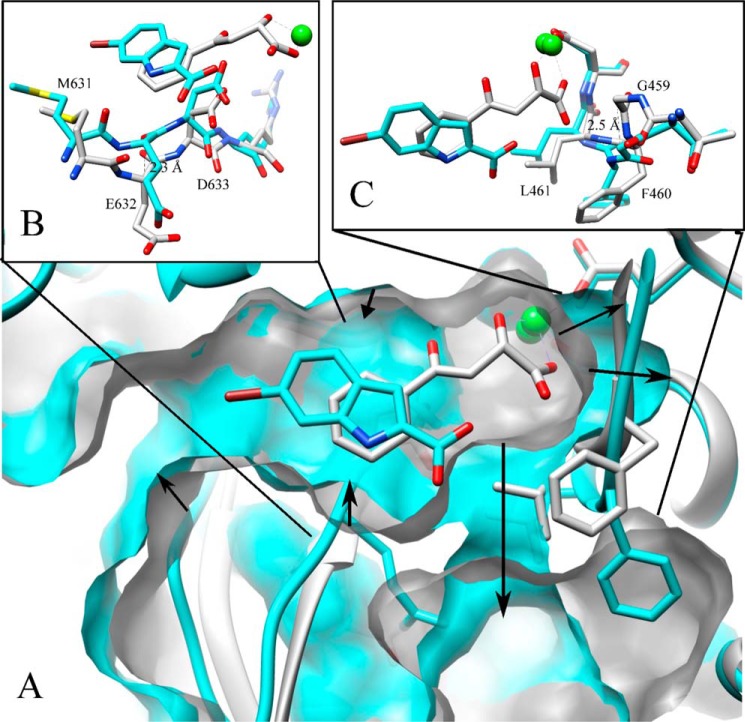
*A*, a close-up of the active site with a superposition of GlcB crystal structures complexed with PDKA (*white*) and fragment **11** (*cyan*) displaying protein surface. The *black arrows* indicate changes upon binding of the Group 2 fragments: the widening of the cavity behind the active site Mg^2+^, the narrowing mouth of the main tunnel caused by the movement of the CoA binding loop (details are shown in *B*), and an additional portal to the solvent with the active site lid opening (details are shown in *C*). Non-carbon atoms are colored as follows: magnesium, *chartreuse*; oxygen, *red*; nitrogen, *blue*; sulfur, *yellow*. Images are rendered in Chimera.

We attempted to determine the crystal structures for all 65 hits complexed with GlcB using both co-crystallization and soaking, however, only 18 fragments resulted in structures. The chemical structures and Δ*T_m_* values of these 18 fragments are shown in Table 1. All 18 structures were determined to relatively high resolution (1.8–2.5 Å) and had clear density for the ligand (example in Fig. 1; omit *F_o_* − *F_c_* difference electron density maps for each fragment shown in supplemental Fig. S1 and Fig. 2). Fragment **10** has an extra electron density peak (∼2.4 σ on the 2*F_o_* − *F_c_* map) within covalent bond distance of carboxylate Cα (supplemental Fig. S2E). The extra density likely arises from an alternative conformation of the carboxylate, but there was insufficient information in the data to build it. In all other cases, observed electron density agreed with the chemical structures of the fragments.

The overall fold of the fragment-bound GlcB structures was similar to those previously determined with glyoxylate (Protein Data Bank code 1N8I), malate and CoA (Protein Data Bank codes 1N8W and 2GQ3), and PDKA inhibitors (11, 20, 24). Domain I is an 8α/8β TIM barrel (residues 115–134 and 266–557), domain II is formed at the C terminus (residues 591–727) and contains mostly helices, and domain III is rich in β-strands and inserted between α1 and β2 of the TIM barrel (residues 135–265). The active site is an ∼20 Å-long tunnel located at the interface of the TIM barrel and a loop comprising residues 616–633 of domain II. A Mg^2+^ ion required for activity is bound at the bottom of the active site tunnel in an octahedral coordination by one oxygen each from the carboxylate side chains of Glu-434 and Asp-462, two water molecules, and two oxygens of glyoxylate. All 18 fragments bind in the active site in positions overlapping with the positions of glyoxylate, malate, and CoA and utilizing many of the interactions responsible for substrate recognition.

The major difference common to all fragment:GlcB complex structures compared with substrate- and inhibitor-complexed is the conformational change in the CoA binding loop (residues 619–633). The CoA binding loop moves “in,” narrowing the active site tunnel by ∼2.5 Å (2.5–2.7 Å measured at the Glu-632 Cα across all fragment complex structures compared to glyoxylate bond structure). Fragment-bound structures display a slight widening of the cavity behind the active site Mg^2+^ ion compared with the GlcB-glyoxylate, GlcB-malate-CoA, and GlcB-PDKA structures. This is caused by the movements of the Glu-434 and Asp-462 side chain carboxylates (0.4–0.5 Å measured at coordinating oxygen atoms) away from the Mg^2+^ ion. Fig. 2 shows a comparison between the “in,” or narrow, and “out” substrate/inhibitor-bound conformations in the GlcB crystal structures.

Although all of the fragment-bound GlcB structures share the “in” CoA binding loop conformation, they show two distinct conformations for the region made by residues 458–462. Five of the hits (Fragments 1–5) show this region in a conformation very similar to the substrate/inhibitor-bound structures. In the other 13 fragment structures, this region, which we now refer to as the active site lid, undergoes a major conformational change. The lid is located at the end of the α-helix corresponding to αH in the *Escherichia coli* MSG structure (Protein Data Bank code 1D8C (25)). It is part of the active site cleft and is in close proximity to the Mg^2+^ ion with one oxygen of the carboxylate of Asp-462 participating in Mg^2+^ coordination. The observed conformational change between closed and open requires the last residue of the αH α-helix (Phe-460) to unwind to a coiled conformation. The backbone carbonyl of Thr-458 moves about 1 Å toward the new opening and swings up 60° toward the Mg^2+^ ion followed by the largest transition in the next two residues, 459 and 460. The Gly-459 backbone rotates ∼70° and moves 3 Å away from the Mg^2+^ ion, and the Phe-460 backbone moves 2.5 Å (measured at the carbonyl oxygen), and its side chain flips by 4 Å (measured at Cγ). The large conformational change of the active site lid ultimately creates a solvent-accessible portal that is ∼7 Å in diameter, distal to the CoA binding tunnel entrance, and very close to the Mg^2+^ ion as shown in Fig. 2.

##### Binding Modes of Fragments

**FIGURE 3.**
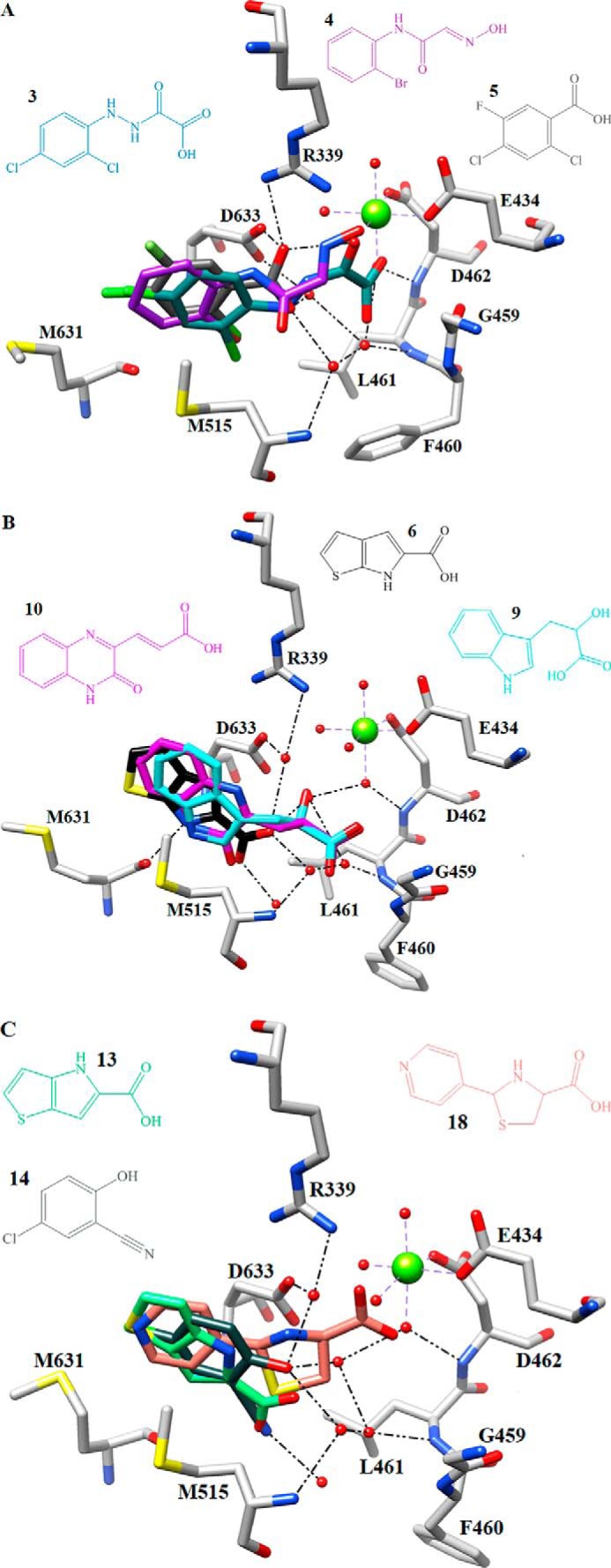
**Superposition of complexed GlcB crystal structures with selected fragments from each group and subgroup showing the hydrogen bond interactions for one representative fragment with protein carbon atoms depicted in *gray*.** *A*, overlay of Group 1 fragments: **3** (*sea green*)-, **4** (*purple*)-, and **5** (*light gray*)-bound GlcB crystal structures, H-bonds, and waters displayed for fragment **5**. *B*, overlay of Group 2.1 fragments: **6** (*black*)-, **9** (*cyan*)-, and **10** (*magenta*)-bound GlcB crystal structures, H-bonds, and waters displayed for fragment **6**. *C*, overlay of Group 2.2 fragments **13** (*green*)-, **14** (*dark slate gray*)-, and **18** (*salmon*)-bound GlcB crystal structures, H-bonds, and waters displayed for fragment **14**. Non-carbon atoms are colored as follows: magnesium, *chartreuse*; oxygen, *red*; nitrogen, *blue*; *chlorine*, *green*; sulfur, *yellow*. Images are rendered in Chimera.

We divided 18 fragments into two main groups based on the conformation of the enzyme to which they bind. Group 1 (fragments **1–5**) bound GlcB structures retain a closed active site lid conformation, and Group 2 (fragments **6–18**) induces an open lid conformation. Group-specific interactions with the active site are shown in Fig. 3. Interactions made by each individual fragment can be examined in supplemental Figs. S3 and S4. All fragments share one prominent interaction with the GlcB active site: aromatic rings bind to a hydrophobic pocket between the side chains of Met-515 and Met-631, in the same manner as the phenyl ring of the PDKA inhibitor. The observation that PDKA with the benzyl ring replaced by cyclohexyl loses its inhibitory activity underlines the importance of these interactions. This part of the active site accommodates a pantothenate group of CoA in the product-bound structures. The rings of most of the fragments in the set contain groups that could interact with the Mg^2+^ such as carboxylates and hydroxyls, but only five fragments (**1–4** and **18**) were observed to directly interact with the Mg^2+^ ion. Although no obvious chemical features distinguish the fragments of Group 1 from Group 2, patterns of protein interactions discriminate between the two groups.

Most Group 1 fragments contribute either one or two oxygens to the coordination of Mg^2+^ much like we observed for glyoxylate and the PDKA inhibitors (Fig. 3*A* and supplemental Fig. S3, A–D). Fragment **5** is the only one in this group that does not directly coordinate Mg^2+^; instead, its carboxylate oxygens hydrogen bond with two water molecules from the Mg^2+^ coordination sphere (supplemental Fig. S3E). Group 1 fragments interact with the side chains of the catalytic residues Asp-633 and Arg-339, which in turn contribute hydrogen bonds to maintain the Mg^2+^ coordination sphere in a second and first order interaction, respectively. With fragments **3, 4,** and **5**, the distance to Asp-633 Oδ is 2.5–2.9 Å, fragment **2** interacts with the backbone nitrogen of Asp-633 at *d*_O-N_ = 3.0 Å, fragment **1** makes van der Waals contact with Oδ at *d*_O-C_ = 3.2 Å, and the distance from the fragments of this group to Arg-339 is *d*_O-Nω_ =2.7–2.9 Å. In addition, all Group 1 fragments interact with the amino acids of the active site lid, further stabilizing the closed conformation. Fragments **2** and **3** directly interact through hydrogen bonds with the backbone nitrogens of Leu-461 and Asp-462 at *d*_O-N_ = 2.9 and 3.0 Å for both fragments. The rest of the group (fragments **1**, **4**, and **5**) hydrogen bond with these residues via ordered water molecules.

Although the Group 2 fragments also maintain their aromatic rings in the hydrophobic pocket, in contrast to Group 1, none of the 13 fragments of Group 2 coordinate Mg^2+^. Fragment **18** binds Mg^2+^ with its acid moiety in a bidentate manner. The distances between the oxygens and the metal longer than optimal for coordinating (2.4 and 2.42 Å). In addition, most of the Group 2 fragments do not hydrogen bond with catalytic residues Asp-633 and Arg-399. Moreover, the majority of Group 2 have no direct interactions with the residues of the active site lid. Instead, ring substituents from this group hydrogen bond primarily with ordered water molecules that gained access through the opening and were not observed in the closed lid conformation of the enzyme. This water-mediated extensive hydrogen bond network appears to stabilize the open lid conformation as shown in Fig. 3, *B* and *C*. For example, the carboxylate oxygen of fragment **7** interacts with a water molecule (hydrogen bond distance, 2.6 Å), which in turn hydrogen bonds to the backbone nitrogens of Phe-460 and Leu-461 (at distances of 3.1 and 2.9 Å) (supplemental Fig. S4B).

The interactions discussed above allowed us to specify which structural features distinguish the two groups of fragments. Most Group 1 fragments have a linker of four to five atoms between the aromatic ring and the Mg^2+^ binding group similar to PDKA inhibitors, which have a four-carbon linker. Given that the hydrophobic interactions with the Met-631 and Met-515 side chains position the aromatic rings of all the ligands in approximately the same part of the active site, the distance between Mg^2+^ and the closest carbon of the fragment ring ranges between 6.3 and 8.4 Å. The four- to five-atom linkers in fragments **1–4** allowed them to successfully span this distance. Although Group 2 fragments contain carboxylate, nitrile, hydroxyl, and diazole groups, all of which could bind to Mg^2+^, these substituents are spaced closer to the aromatic ring than in Group 1. For seven of the 13 Group 2 fragments (**6–8**, **11**, **13**, **14**, and **17**), it is only one carbon away from the aromatic ring. Hence, it appears that the spacing and positioning of potential hydrogen-bonding and/or Mg^2+^-coordinating substituents on the aromatic moiety of the ligand and not their nature define their preference for a particular enzyme conformation.

##### Observations from GlcB-Fragment Complex Structures for Future Inhibitor Design

Halogen substituents on the aromatic ring appear to be beneficial for binding affinity. For example, benzoic acid with three halogen substituents (5) gives a 3.3 °C shift in melting temperature, whereas neither single fluorine-substituted nor doubly chlorine- and methyl group-substituted benzoic acid binds. Another feature enhancing the interaction of the aromatic moiety with this hydrophobic pocket is the hydrogen bond donor included in the ring or ring system. Seven of 13 fragments in Group 2 were separated into subgroup 2.1 as they all make a hydrogen bond with the backbone carbonyl of Met-631 (*d*_N-O_ = 2.6–3.2 Å; Fig. 3*B* and supplemental Fig. S4, A–G), resulting in a relatively high Δ*T_m_* ranging between 4.6 and 8.6 °C.

Interestingly, the crystal structure of GlcB in complex with **14** indicated two molecules of the fragment bound to the active site (supplemental Fig. S4I). Fragment **14** is a trisubstituted phenyl moiety with nitrile, hydroxyl, and chloro substitutions at the 1-, 2-, and 5-positions, respectively. One molecule of **14** binds in the hydrophobic pocket of the active site similarly to the other fragments, forming hydrogen bonds with three ordered water molecules via its hydroxyl oxygen and a fourth water molecule with its nitrile nitrogen (hydrogen bond distances, 2.7–3.0 Å). The other fragment binds 4.1 Å away near the entrance of the tunnel in the hydrophobic pocket formed by Pro-120, Phe-126, and Met-631 and interacts with one water molecule via a hydrogen bond to its hydroxyl oxygen (2.8 Å). This is the only molecule from the set which binds far from the Mg^2+^, to the active site tunnel entrance where the adenine ring of acetyl-CoA binds. The information about interactions can be used for extending inhibitors into that part of the active site in the future.

Fragment **15** also presents interactions along the path the CoA pantetheinyl moiety follows in the active site. The fragment is phenyl-substituted with a thioacrylate tail, which provides a linker long enough to reach from the hydrophobic pocket to the Mg^2+^. It is similar to fragment **1** without the chlorine on the ring and with a double bond in the linker. However, the crystal structure of the **15**-bound GlcB (supplemental Fig. S4J) shows that the thioacrylate tail actually points in the opposite direction from the Mg^2+^ with one of the carboxylate oxygens forming a hydrogen bond with a water molecule and both carboxylate oxygens forming van der Waals interactions with the backbone carbonyls of Pro-543 and Met-631 (2.6 and 3.0 Å, respectively). The relatively large and hydrophobic sulfur atom and the double bond in the linker is the likely cause of this positioning in the active site; it favors the hydrophobic side of the tunnel over the highly polar Mg^2+^ surroundings.

Fragment **18** belongs to Group 2 because it binds to the open lid form, but it has the full set of interactions characteristic of Group 1. It interacts with both catalytic residues, Asp-633 and Arg-339: the nitrogen and two carbons of the thiazolidine ring engage the carboxylate oxygen of the Asp-633 side chain via hydrogen bond (*d*_N-O_ = 3.1 Å) and forms van der Waals interactions (*d*_C-O_ = 3.1 Å), and one of its carboxylate oxygens forms a hydrogen bond with one of the Arg-339 side chain nitrogens (*d*_O-N_ = 2.7 Å). Moreover, its two carboxylate oxygens interact with Mg^2+^ in a bidentate manner at distances of 2.3 and 2.4 Å, which are longer than ideal for metal coordination. It is similar to Group 1 fragment **1** but different from the rest of the fragments in Group 2 as their carboxylates do not interact with Mg^2+^ (supplemental Fig. S4M). This information suggests that interactions with one of the two enzyme conformations are not necessarily mutually exclusive and can be combined in future inhibitor designs. Altogether, the fragment structures we report provide a wealth of information about the preferred chemotypes and potential interactions to be exploited along ∼16 Å of the main active site tunnel plus the additional portal created by the active site lid opening.

##### Fragment Binding Information Used in Inhibitor Design

**FIGURE 4.**
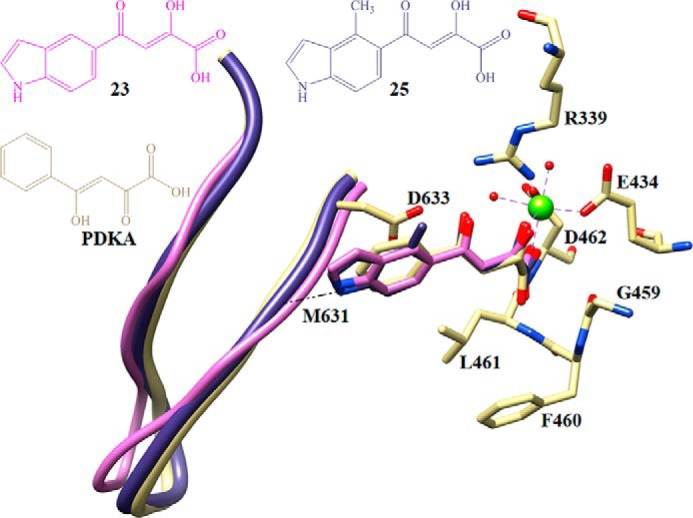
**Overlay of crystal structures of GlcB complexed with PDKA and indole diketo acid inhibitors. 23**, *pink*; **25**, *slate blue*; PDKA, *yellow*. The CoA binding loop position is shown in *ribbons*. Non-carbon atoms are colored as follows: magnesium, *chartreuse*; oxygen, *red*; nitrogen, *blue*; sulfur, *yellow*. Images are rendered in Chimera.

To test how the information from fragment binding can be used to design inhibitors or to improve existing ones, we attempted to incorporate new interaction discovered through our fragment:GlcB complex structures into the existing PDKA inhibitors. Eight of 13 fragments in Group 2 (**6–12**) form hydrogen bonds with the backbone carbonyl of Met-631 through a nitrogen on a five-membered pyrrole moiety (**8**) or a fused indole, thienopyrrole, or quinoxaline moiety (**6**, **7**, and **9–12**) (*d*_N-O_ = 2.6–3.5 Å; Fig. 3*B* and supplemental Fig. S4, A–G). Because the Group 2 fragments bind to a different enzyme conformation compared with PDKA, we didn't rely on a superposition of the complex structures to choose the ring attachment position but rather explored multiple substitution positions to combine hydrogen bond-forming aromatic ring with the diketo acid group. This resulted in the series of compounds presented in Table 2, which lists the indole-based inhibitors and their potencies against the enzyme. Compounds **19–21** were designed based on the indole-containing fragment **9** with the diketo moiety appended at the 3-position of the indole ring. Compound **22** was based on fragment **11** with the diketo acid moiety at the 2-position of the indole ring. In the crystal structure of GlcB in complex with **19**, we observed a weak hydrogen bond between the indole and the backbone carbonyl oxygen of Met-631 (*d*_N-O_ = 3.2 Å) with the CoA binding loop in the “in” conformation similar to the fragment:GlcB complex structures as shown in Fig. 4. We expanded the series by attaching the diketo acid moiety at the 5-position of the indole, resulting in the indole diketo acids **23–27**. Of the five inhibitors, **23** showed the highest potency with an IC_50_ of 20 nm, and its analogue, **25**, with a 4-position methyl substitution for stability improvement was the second best with an IC_50_ of 170 nm. It is interesting that addition of a methyl group to position 3 on the indole ring of **23** (compound **24**) resulted in significant loss of inhibitory activity. Based on the modeling using the GlcB-**23** complex crystal structure, the most probable cause of this loss is the potential overlap of the methyl group with the backbone carbonyl of Val-118. This region of the binding pocket is tightly packed, and we believe that the 3-methyl could not be accommodated.

**TABLE 2:**
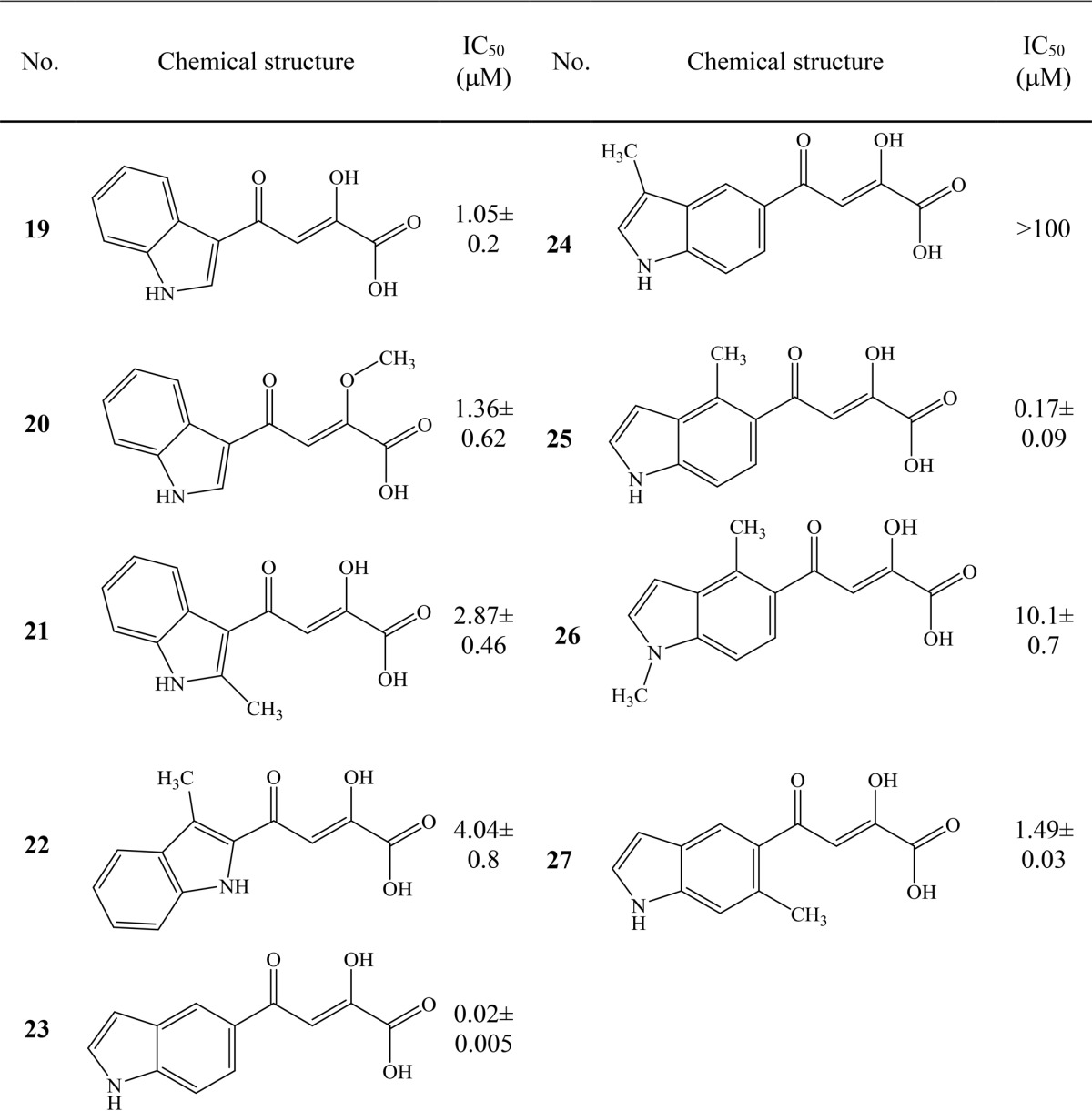
**Enzyme activity data for fragment based indole diketo acid inhibitors** Standard deviations are calculated based on two independent measurements for **19–22**, **24**, **26**, and **27**; for **23** and **25**, five measurements have been performed.

We solved structures of malate synthase in complex with these inhibitors to examine their interactions with the enzyme. The indole containing PDKAs were observed to make strong hydrogen bonds between the indoles and backbone carbonyl of Met-631 in the complex crystal structures with **23** and **25** (*d*_N-O_ =2.7 and 2.4 Å, respectively) as shown in Fig. 4. The CoA binding loop has the “in” conformation in the **23** complex crystal structure. Interestingly, for **25**, the CoA binding loop is halfway between the “in” and “out” conformation in the complex crystal structure (1.5 Å distance measured between the Glu-632 Cα and the Glu-632 Cα of PDKA when overlaying complex GlcB structures) as shown in Fig. 4. All three inhibitor complex crystal structures, **19**, **23**, and **25**, have a closed active site lid, similar to PDKA, due to the interactions of the diketo acid moiety with the closed active site lid residues (contacts for each of them are shown in supplemental Fig. S5). The successful incorporation of an additional hydrogen bond and induction of the anticipated conformational change in the active site underlines the great potential that fragment binding information holds for future drug design.

##### Preincubation with Substrates Leads to Apo and GlcB-Product Complex Crystal Structures

The open conformation of the active site lid has previously been observed in the structure of malate synthase isoform A from *E. coli* (26) in which only a Mg^2+^ ion was bound in the active site of the enzyme. It is similar to the conformation we found in the fragment-bound structures: the opening of the active site lid is concurrent with the narrowing of the main channel due to the CoA binding loop moving to the “in” conformation. Although this is a different isoform of malate synthase from that of *M. tuberculosis* (A *versus* G) with a relativity low sequence identity of 18–20% (20), all the catalytic residues and the residues responsible for stabilizing the “in” and “out” conformations of the CoA binding loop and the “closed” and “open” conformations of the active site lid are conserved. There were two distinct conformations of the enzyme throughout all fragment-bound structures. Taken together it led us to hypothesis that these conformational changes are physiologically relevant and prompted us to further investigate the structural changes associated with the reaction cycle of malate synthase.

Because the published structures of substrate- and product-bound GlcB are virtually identical, the enzyme was never viewed as undergoing conformational changes upon catalysis. With fragments as probes, we were able to capture more transient states assumed by GlcB during turnover. Although all published structures agree on the conformation of the enzyme, two different structures of *M. tuberculosis* GlcB in complex with malate and CoA are reported (Protein Data Bank codes 1N8W (20) and 2GQ3 (24)). In the first structure from Smith *et al.* (20), malate contributes hydroxyl oxygen and one of the carboxylate oxygens to the Mg^2+^ coordination sphere (distorted pyramidal) along with Glu-434, Asp-462, and one water molecule (distances, 1.8–2.5 Å). In the second structure from Anstrom and Remington (24), malate essentially overlaps with the glyoxylate position, giving Mg^2+^ octahedral coordination (distances, 2.0–2.2 Å). The CoA-bound crystals are of relatively poor quality compared with the glyoxylate-bound crystals. As it is more challenging to build in a low resolution electron density map, the position of malate in the 1N8W model was questioned in the publication by Anstrom and Remington (24).

The 1N8W structure was solved from a crystal formed after the enzyme had been preincubated with the substrates and hence captured the products, whereas for the 2GQ3 structure GlcB was mixed with products prior to crystallization. Because the protonation states of malate and the catalytic residues would differ between these two methods, we believe both malate-bound structures are correct. We have reproduced the 1N8W structure from Smith *et al.* (20) by following the published protocol and preincubating wild-type GlcB with equal concentrations of substrates glyoxylate and AcCoA (2 mm each) for 20 min prior to setting up the crystal plate. The data (best set at 2.5 Å resolution) showed electron density consistent with the position of malate in the 1N8W structure. The position of CoA differs slightly from 1N8W, which is in agreement with previous observations (20, 24–26) that after turnover CoA does not have well ordered density.

**FIGURE 5.**
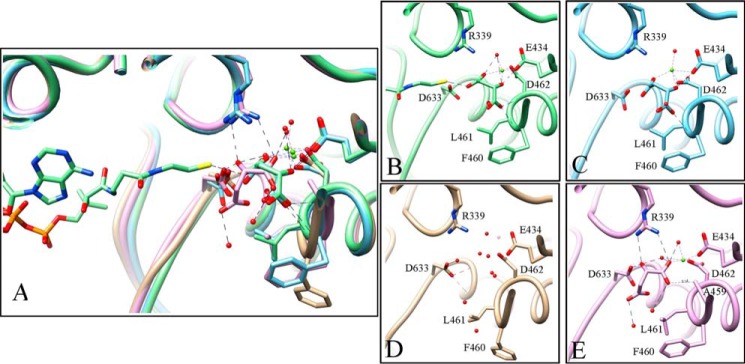
**Positions of malate in model 1N8W and in the crystal structures from GlcB preincubated with substrates at various times.** *A*, overlay of different positions of malate in model 1N8W (*light green*; *B*), malate-bound structure after 40-min preincubation (*light blue*; *C*), apoenzyme resulting from preincubation at 60 min (*gold*; *D*), and G459A mutant complex with malate after preincubation with substrates (*pink*; *E*). Non-carbon atoms are colored as follows: magnesium, *chartreuse*; oxygen, *red*; nitrogen, *blue*; sulfur, *yellow*. Images are rendered in Chimera.

Crystals produced after preincubating GlcB with substrates glyoxylate and AcCoA for 30, 40, 50, and 60 min prior to crystallization were examined for product. Multiple crystals were examined for each time point with ∼20 total data sets collected and analyzed. Crystals from 30-, 40-, and 50-min preincubations gave ambiguous electron densities with only partial electron densities for CoA and the Asp-462 side chain present. The enzyme crystallized after longer preincubation with the substrates (60 min) resulting in a refined model of the GlcB-malate complex. No density for CoA was observed, and malate assumed a similar position to the malate of the 1N8W model (Fig. 5). The Asp-462 side chain has less defined electron density and its B factors changed from 8–12 in the CoA-malate-bound structure 1N8W (average B factor for the chain is 62.9) to 52–98 in the malate only-bound structure (average B factor for the chain is 78.9), and the Mg^2+^ B factor increased from 40 to 76.56. The active site loop is closed as malate still maintains a weak hydrogen bond with the backbone nitrogen of Leu-461 (3.0 Å) but is too far to hydrogen bond with the Phe-460 backbone (3.5 Å).

Based on our hypothesis that the active site loop opens to allow for product/substrate exchange, we made a G459A mutant as Gly-459 underwent the largest main chain movement upon lid opening. The additional methyl group of the alanine side chain likely restricted the movement as anticipated, and the mutant showed 20% activity when tested in the enzyme assay. The crystal produced after preincubation with substrates belonged to the P4_3_2_1_2 space group and diffracted to 2.7 Å resolution. As expected, it had no density for CoA as the packing of the enzyme in this space group is not compatible with CoA binding, and had malate in the active site at a position distinct from that observed in the 1N8W structure. The crystal structure was particularly interesting because it showed that Asp-633 had drawn closer to the Mg^2+^, making interactions with the oxygens of malate carboxylate (*d*_OD1-O5_ = 2.8 Å) and hydroxyl (*d*_OD2-O3_ = 2.5 Å). This hydroxyl is also within hydrogen bond distance to Arg-339 (*d*_O-Nω_ = 3.0 Å). Asp-633 movement places the conformation of the CoA binding loop in a position between “out” and “in,” 1 Å from each as measured at Glu-632 Cα. The Mg^2+^ is in a pyramidal coordination with two water molecules, Glu-434, Asp-462, and one malate carboxylate oxygen. The other oxygen on the malate carboxylate is making contact with the mutated Ala-459 side chain with van der Waals interactions (3.1 Å) as shown in Fig. 5.

Finally, we obtained crystals of ligand-free GlcB to prove that the open lid protein conformation discovered through fragment is indeed corresponding to the apo form of the enzyme. The best quality malate synthase crystals are packed in the P4_3_2_1_2 space group and always have density for bound glyoxylate, which tends to co-purify with the enzyme. We diluted the enzyme to 0.1 mg/ml in assay buffer containing 5 mm Mg^2+^ and incubated it with substrates to remove residual glyoxylate. After 1 h, we concentrated the enzyme back to 5 mg/ml, extensively washing it with an excess of the same buffer. This sample yielded a P4_3_2_1_2 space group crystal that diffracted to 2.2 Å, showing well defined density for the CoA binding loop in the “in” conformation and an open active site lid. This is consistent with the conformation observed for the Group 2 fragments and was achieved with no ligand bound in the active site. Interestingly, there is no electron density for Mg^2+^; the peak at the corresponding position is of the similar intensity to surrounding waters (2 σ in the 2*F_o_* − *F_c_* map) (Fig. 5). Collection of multiple crystals were required to achieve this uniform conformation of the moving loop structure with the data still of lesser quality than the glyoxylate-bound P4_3_2_1_2 crystals used for fragment soaking. However, using fragments, we were able to reliably obtain high resolution data using good quality preformed crystals and to achieve more transient state structures by soaking fragments, to stabilize these enzyme conformations.

## Discussion

We used fragments, which are relatively small molecules (molecular weight <300), to probe the active site of *M. tuberculosis* malate synthase for possible binding modes. The GlcB-fragment complex crystal structures have given us several novel findings including an additional portal from the active site to the surface of the enzyme. These structures allowed us to analyze the sequence of conformational changes of both the active site lid (residues 458–462) and the CoA binding loop (residues 619–633). We propose that the observed conformations represent the enzyme's catalytic cycle: the active site lid change between the open and closed forms and the CoA binding loop transition between the “in” and “out” positions. The mechanism and order of the reaction for malate synthase have been previously determined by isotope exchange experiments (27) and by using dead-end product inhibition and kinetic isotope effects (28). The results showed that glyoxylate binds first followed by the binding of AcCoA. A proton is abstracted from the acetyl group by an active site base, Asp-633, and the C–C bond is formed between activated acetyl and glyoxylate, which is polarized by Mg^2+^. Subsequently the malyl-CoA intermediate is hydrolyzed into malate and CoA. Based on solvent isotope exchange experiments, Quartararo and Blanchard (28) further proposed that one of the Mg^2+^-coordinated water molecules is used in the hydrolysis of malyl-CoA and that the active site acid, Arg-339, donates a hydrogen to the SH leaving group of CoA.

Two product-bound structures of *M. tuberculosis* GlcB have been published with CoA occupying similar positions but differing in malate position. In the structure by Smith *et al.* (20), malate binds by coordinating the Mg^2+^ ion in a distorted pyramidal geometry. In the structure by Anstrom and Remington (24), the position of malate overlaps with the position of glyoxylate in the substrate-bound structure, and the Mg^2+^ ion is coordinated by malate, Glu-434, Asp-462, and two water molecules in a perfect octahedral geometry (24). The latter structure was obtained by co-crystallizing GlcB with malate and CoA. We repeated the experiment following the method of Smith *et al.* (20), crystallizing GlcB after incubation with substrates and capturing the products formed in the active site. Our result reproduced 1N8W structure with malate's binding conformation as a part of the distorted pyramidal coordination of the Mg^2+^ ion. This conformation agrees with the mechanism proposed by Quartararo and Blanchard, with one of the Mg^2+^ coordination sphere waters being used for hydrolysis of the intermediate (28). Moreover, we observed the later stages of the catalytic cycle in the malate-bound structure after CoA dissociation, and we obtained the complete GlcB apo structure in crystals from the longest incubation with an excess of substrates in the reaction mixture. The apo structure has the “in” conformation for the CoA binding loop and the open active site lid conformation originally discovered through the fragment-bound structures, with a disordered Mg^2+^ ion.

Combining our observations from all the crystal structures and published kinetic studies thus far, we propose that glyoxylate binds first to the apo enzyme (active site lid open and CoA binding loop “in”). Upon making two strong hydrogen bonds with the backbone nitrogen atoms of Glu-434 and Asp-462, it induces the closing of the active site lid. Subsequently, glyoxylate brings order to the Mg^2+^ coordination sphere, enabling the octahedral geometry, and the hydrogen bond network surrounding the Mg^2+^-glyoxylate complex pushes Asp-633 back to establish the CoA binding loop in the “out” conformation. This widens the main tunnel sufficiently for the pantothenate tail of AcCoA to enter and bind productively. Once the reaction is complete, CoA dissociates, and Asp-633 loses the hydrogen bond with the malyl-CoA intermediate. In the active site where the Mg^2+^ coordination sphere and the catalytic acid-base pair are shielded from solvent, the catalytic turnover changes the hydrogen bond network in response to the changes in the protonation states of the participants. The side chain of Asp-633 can not form a hydrogen bond with malate because both of the carboxylate groups are proposed to be protonated at this point (27, 28). Asp-633 then moves toward Mg^2+^ and displaces malate so that the malate carboxylate group is within hydrogen bonding distance of both the Asp-633 and Arg-339 side chains (as observed in the malate-bound structure of the catalytically hindered G459A GlcB mutant), possibly serving as a hydrogen relay to restore the Asp-633 and Arg-339 protonation states for the next round. The malate hydroxyl group binds in place of the Mg^2+^-coordinating glyoxylate carbonyl group, and one of the Mg^2+^ sphere water molecules is lost to hydrolysis of the malyl-CoA intermediate. This destabilizes the octahedral coordination of Mg^2+^, which reflects on the position of the Asp-462 side chain as it participates in this coordination sphere. This destabilization causes the end of the α-helix to unwind, starting from the Mg^2+^-coordinating Asp-462 down to Gly-459. In addition, the hydrogen bond between the Asp-462 backbone nitrogen and carbonyl of the glyoxylate cannot be formed with the malate hydroxyl, further favoring the opened conformation. As a result, the active site lid opens with the largest main chain transition at Gly-459, and the bulky side chains of Phe-460 and Leu-461 relocate to create a new solvent access portal. It is through this portal that malate leaves and a new glyoxylate enters for the next round of catalysis.

The two conformations of the CoA binding loop are stabilized by the key residues Glu-632 and Thr-636, which are conserved in malate synthase homologs across different species of bacteria including *Corynebacterium glutamicum* and *E. coli* (both A and G isoforms). As the CoA binding loop changes from the “in” to the “out” conformation, widening the tunnel by ∼2 Å, the side chain of Glu-632 moves more than 3.5 Å, and the side chain of Thr-636 rotates more than 180° to retain the hydrogen bond formed between the two residues.

The open conformation of the active site lid has previously been observed in the *E. coli* malate synthase A (MSA) (26). *M. tuberculosis* only has the G isoform, and it shares 56% identity and 71% similarity with *E. coli* MSG but only 18% identity with the MSA isoform. Lohman *et al.* (26) proposed that the active site lid open conformation is related to substrate/product exchange during catalysis. Although the *E. coli* MSA isoform has residues Arg-276 and Trp-277 in place of *M. tuberculosis* GlcB Phe-460 and Leu-461, they serve a similar purpose of closing the entrance of the second portal with their bulky side chains. Meanwhile, the transition of the active site lid main chain from closed to open remains the same, and Gly-459, which is conserved across both isoforms, sustains the most significant twist with its smallest hydrogen side chain. The side chain of nearby residue Tyr-492 forms a hydrogen bond with the backbone nitrogen atom of Gly-459 regardless of the active site loop conformation. All of these residues, Gly-459, Tyr-492, Glu-632, Ala-635, and Thr-636, are conserved not only across different species of GlcB but across two isoforms of GlcB as well, supporting the view that these two conformations are physiologically relevant.

Using fragment based methods we discovered additional conformations which we propose the enzyme assumes throughout catalysis. It represents a utility of fragment-based screening: fragment hits used as stabilizers of transient protein conformations, making these conformations amenable to study with structural biology methods. Preferred binding chemotypes and active site information that is not limited by one conformation can increase the chance of success of computational and medicinal chemistry efforts. Alternative conformations of the active site lid and the CoA binding loop can be achieved independently of each other as demonstrated by our crystal structures complexed with **19, 23**, and **25** and thus can be exploited in any combination for inhibitor design. Several fragment hits have nitrogen-containing groups such as pyrrole, indole, thienopyrrole in which the nitrogen interacts with the backbone carbonyl of Met-631. We have incorporated an indole into our existing inhibitor series. As a result, we have increased potency by over 100-fold (IC_50_ values of 20 nm for indole diketo acid *versus* 2 μm for parent PDKA) and observed the anticipated hydrogen bond between the ring nitrogen and Met-631 carbonyl. These key conformational changes, along with the information about the interactions between the enzyme and fragments, will aid in the structure based drug design efforts on malate synthase.

## Experimental Procedures

### 

#### 

##### Cloning and Purification

The wild-type, C619A mutant, or C619A,G459A double mutant of *M. tuberculosis* GlcB (Rv1837c) were cloned into a custom vector, p6HisF11d, and expressed in *E. coli* BL21 cells with an N-terminal His tag. Upon harvesting, the cells were pelleted and stored at −20 °C. Cells were resuspended in buffer containing 20 mm Tris-HCl, pH 7.5, 100 mm NaCl, and 5 mm imidazole, and purified by nickel affinity column (HisTrap FF, GE Healthcare) followed by size exclusion chromatography (HiLoad Superdex 200, GE Healthcare) as described previously (20). The protein was concentrated to 5–10 mg/ml in 20 mm Tris-HCl, pH 7.5, and stored at −80 °C. Because the presence of the His tag did not affect protein unfolding, enzyme activity, or the crystallization process, all of the reported work was done with the GlcB N-terminal His tag intact.

##### DSF Thermal Shift Binding Assay

RT-PCR (Stratagene Mx3005P, Agilent) and SYPRO Orange (Invitrogen, Life Technologies) were used to conduct a DSF assay to screen two fragment libraries against GlcB. The fragments were assembled in 96-well plates and dissolved in 100% DMSO to ∼100 mm concentration by using the average molecular weight of 80 fragments in each plate for the calculation. The total assay volume was 20 μl in each well of the 96-well PCR plates. Solutions of 4–5.5 μl of 4.5–30 μm GlcB in AB buffer (Tris-HCl, pH 7.5, MgCl_2_, and EDTA), 14 μl of 7× SYPRO orange in 600 mm HEPES buffer, pH 7.5, and 0.5–2 μl of 100 mm fragments were added to each well. DMSO was used as a negative control, and the inhibitor 2-bromo-PDKA was used as a positive control. The final concentrations of GlcB, SYPRO Orange, and fragments were 1.5–10 μm, 4.5×, and 2.5–10 mm, respectively. The final AB buffer contained 20 mm Tris-HCl, 5 mm MgCl_2_, and 0.8 mm EDTA, and the final concentration of HEPES buffer, pH 7.5, was 200 mm. Prior to running RT-PCR, the assay plate was sealed with optical PCR thermal film and centrifuged at 1000 rpm for 5 min. For the assay, the plate was equilibrated at 25 °C for 5 min and heated from 25 to 85 °C with a heating rate of 0.5 °C/min by RT-PCR. Fluorescence of SYPRO Orange was monitored in the RT-PCR instrument using the wavelengths 491 and 610 nm for excitation and emission, respectively.

##### Enzyme Activity Assay

CoA produced by the reaction of GlcB was quantified by using Ellman's reagent, 5,5′-dithiobis(2-nitrobenzoic acid), continuously monitoring coupled reaction absorbance at 412 nm for 20 min as described previously (11, 29). The assay was conducted in a 96-well clear plate in a 100 μl reaction total volume with final concentrations of 13 nm GlcB, 20 mm Tris, pH 7.5, 5 mm MgCl_2_, and 0.8 mm EDTA. Compounds were dissolved in 100% DMSO and preincubated with the protein for 20 min prior to starting the reaction at 2% DMSO final concentration. Then acetyl-CoA was added to a final concentration of 0.6 mm, and the reaction was started by addition of 1.2 mm glyoxylate mixed with 0.5 mm 5,5′-dithiobis(2-nitrobenzoic acid) (final concentrations).

##### Compound Synthesis

Chemical syntheses of all compounds used in this study are described in supplemental Table S2 and Scheme S1 .

##### Protein Crystallization, Data Collection, and Data Analysis

GlcB crystals were obtained by hanging drop vapor diffusion within 1–2 months using the previously described method (11, 20). Briefly, purified C619A GlcB (with His_6_ tag) at a concentration of 5 mg/ml in 20 mm Tris-HCl, pH 7.5, was mixed with 20–30% PEG 3350, 0.1 m MgCl_2_, and 0.1 m Tri-HCl, pH 7–8.5 at a 1:1 volume ratio for crystallization. For co-crystallization, a 1–4 mm concentration of the fragment was added to GlcB prior to mixing with mother liquor. Fragments were soaked in by transferring preformed crystals or co-crystals into drops with 15–50 mm fragment in the mother liquor with a final concentration of DMSO below 15% for 16–48 h. The fragment-bound GlcB crystals belonged to space group P4_3_2_1_2 with unit cell dimensions of *a* = *b* = 79 Å, *c* = 224 Å, and α = *b* = γ = 90° and contained one molecule per asymmetric unit. For malate and CoA product-bound crystals, purified wild-type GlcB (∼5 mg/ml) was preincubated for 20, 40, and 60 min with 2 mm substrates glyoxylate and AcCoA each in 20 mm Tris-HCl, pH 7.5, and 5 mm MgCl_2_ at room temperature. The protein mixture was then mixed at a 1:1 volume ratio with 1.6 m (NH_4_)_2_SO_4_, 0.1 m MES, pH 6.5, and 10% dioxane. These crystals belonged to space group P4_1_2_1_2 with unit cell dimensions of *a* = *b* = 121 Å, *c* = 233 Å, and α = *b* = γ = 90° and contained two molecules per asymmetric unit. All crystals were cryoprotected by Fomblin (Sigma) and flash frozen in liquid nitrogen for X-ray data collection. Data were collected at the Argonne National Laboratory Advanced Photon Source synchrotron, beamlines 19-ID and 23-ID, or Lawrence Berkley National Laboratory Advanced Light Source, beamlines 8.2 and 8.3, at 0.98–1.01 Å wavelengths. Diffraction data were indexed, integrated, and scaled using HKL2000 (30) or HKL3000 (31) and were further merged and truncated in CCP4 (Collaborative Computational Project 4) (32). Protein Data Bank structures 1N8I and 1N8W (20) with only the protein atoms remaining in the refinement were used as the starting models for the initial rigid body refinements of the isomorphous P4_3_2_1_2 and P4_1_2_1_2 crystals, respectively, in REFMAC (33). The ligand model and dictionary files were created using ELBOW from the PHENIX suite (34) and fitted into electron density in Coot (35). The final fragment-bound and product-bound models were gradually improved by repeating the process of manual inspection and modification in Coot and refinement in PHENIX. Supplemental Table S3 contains data collection and refinement statistics as well as Protein Data Bank deposition codes for all structures.

## Author Contributions

I. V. K. and J. C. S. conceived the idea of the project. H.-L. H. conducted the fragment screening and obtained crystal structures of complexes with fragments and an inhibitor. I. V. K. obtained the structures of complexes with inhibitors as well as GlcB complexed with products and apo structures. Enzyme purification and activity assays were done by H.-L. H. and I. V. K. Indole compounds were synthesized by V. B. J. and M. K. P. The manuscript was prepared by H.-L. H., I. V. K., and J. C. S.

## Supplementary Material

Supplemental Data

## Footnotes

4 S. Puckett, C. Trujillo, Z. Wang, H. Eoh, T. Ioerger, I. Krieger, J. Sacchettini, D. Schnappinger, K. Y. Rhee, and S. Ehrt, manuscript submitted for publication.

5 T. Ioerger, I. V. Krieger, and J. C. Sacchettini, unpublished data.

## Footnotes

3 The abbreviations used are:

PDKA: phenyl-diketo acid
GlcB: malate synthase
MSA: malate synthase A
MSG: malate synthase G
AcCoA: acetyl coenzyme A
CoA: coenzyme A
ICL: isocitrate lyase
DSF: differential scanning fluorimetry
*T_m_*: melting temperature.
