# Supplementary material for: Mycobacterium tuberculosis Malate Synthase Structures with Fragments Reveal a Portal for Substrate/Product Exchange
Source: J Biol Chem. 2016 Oct 13;291(53):27421–32. doi: 10.1074/jbc.M116.750877 (PMC5207166; doi:10.1074/jbc.M116.750877)
Supplement: Supplemental Data [file supp_291_53_27421__index.html]

Mycobacterium tuberculosis Malate Synthase Structures with Fragments Reveal a Portal for Substrate/Product Exchange — Mycobacterium tuberculosis Malate Synthase Structures with Fragments Reveal a Portal for Substrate/Product Exchange — Probing Malate Synthase from M. tuberculosis with Fragments — Supplemental Data 

# *Mycobacterium tuberculosis* Malate Synthase Structures with Fragments Reveal a Portal for Substrate/Product Exchange

## Supplemental Data

- Supplemental information (.pdf, 2.7 MB) - File containing all supplemental tables, figures and information
