# Supplementary material for: Mycobacterium tuberculosis Malate Synthase Structures with Fragments Reveal a Portal for Substrate/Product Exchange
Source: J Biol Chem. 2016 Oct 13;291(53):27421–32. doi: 10.1074/jbc.M116.750877 (PMC5207166; doi:10.1074/jbc.M116.750877)

Supplemental Data

for

Mycobacterium tuberculosis malate synthase structures with fragments reveal a portal for substrate/product exchange

Hsiao-Ling Huang[1*], Inna V. Krieger[2*], Maloy K. Parai[2], Vijay B. Gawandi[2], James C. Sacchettini[1,2]

[1]Department of Chemistry

[2]Department of Biochemistry and Biophysics

Texas A&M University, College Station, Texas 77845, USA

Table S1. Chemical structure and ΔT_m for hit (no crystal structure) from the fragment screen.

Chemical Structure	ΔT_m (°C)	Chemical Structure	ΔT_m (°C)
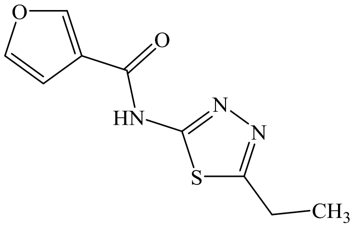	3.3	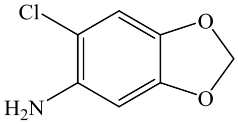	3.3
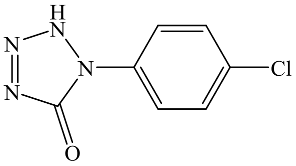	3.6	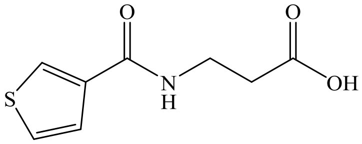	3.1
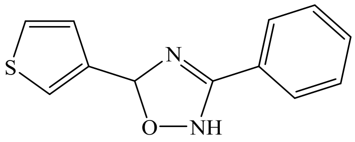	4.6	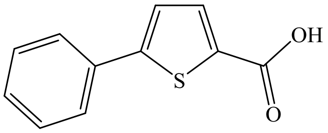	3.4
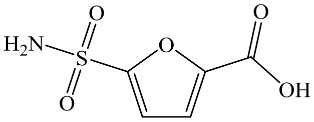	3.3	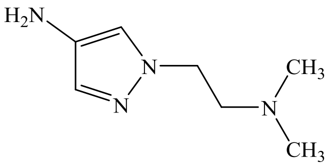	3.2
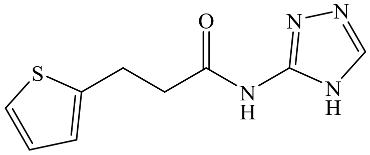	3.2	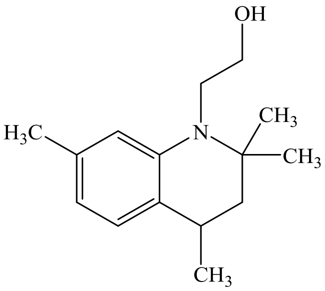	3.9
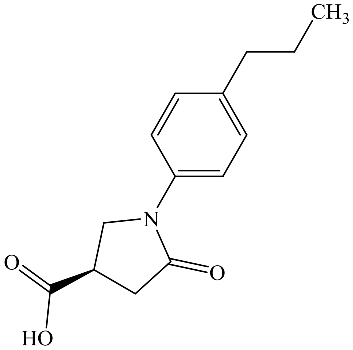	3.7	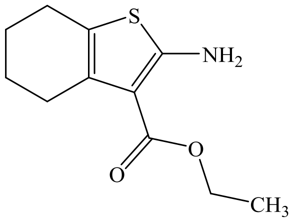	4.5

Table S1. Continued.

Chemical Structure	ΔT_m (°C)	Chemical Structure	ΔT_m (°C)
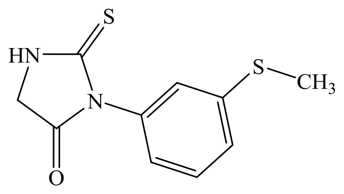	3.2	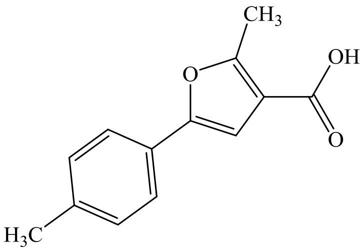	6.9
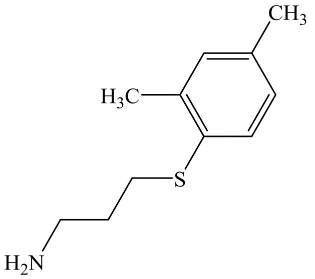	4.9	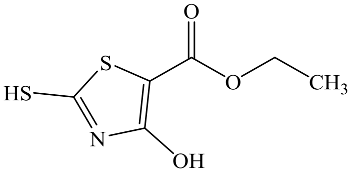	3.1
		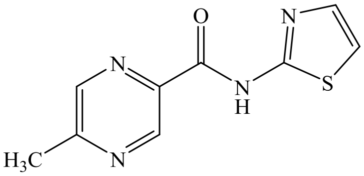	3.4
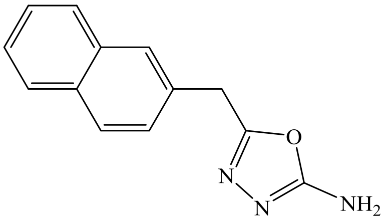	3.1	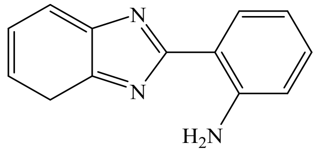	4.3
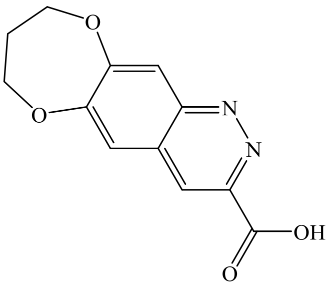	3.2	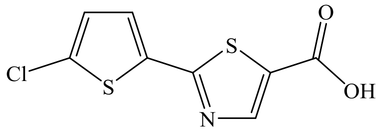	3.4
		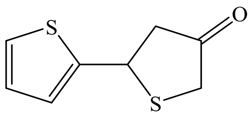	3.3
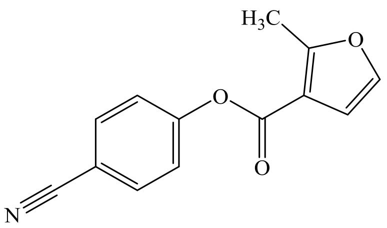	3.3	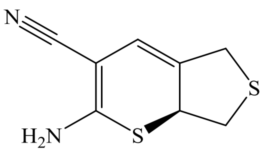	4.3

Table S1. Continued.

Chemical Structure	ΔT_m (°C)	Chemical Structure	ΔT_m (°C)
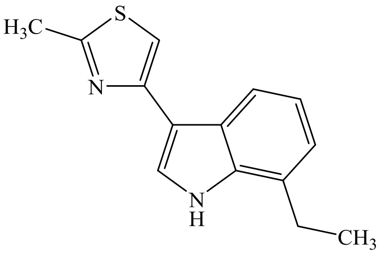	3.6	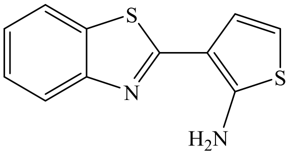	5.8
		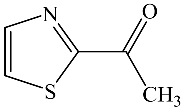	3.5
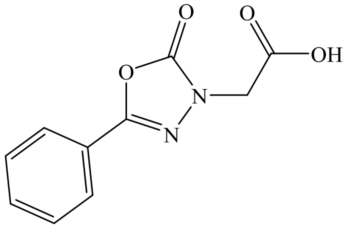	3.5	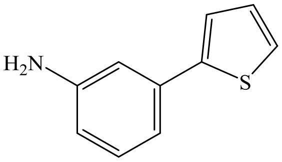	3.4
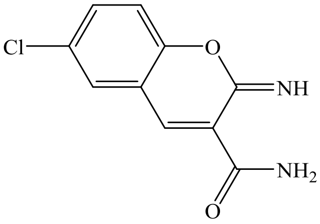	3.5	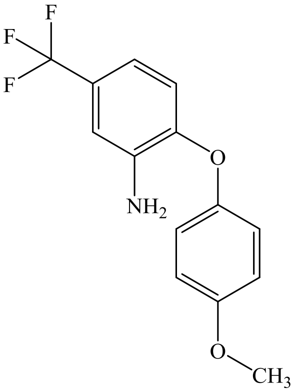	6.5
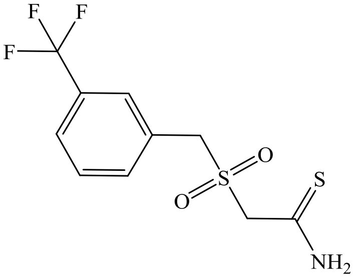	5.7	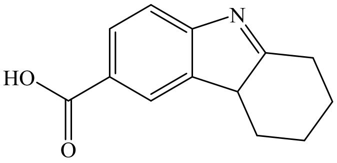	3.4
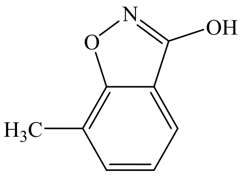	3.5	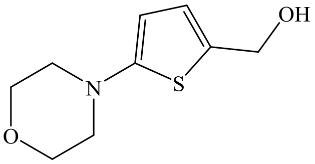	6.9

Table S1. Continued.

Chemical Structure	ΔT_m (°C)	Chemical Structure	ΔT_m (°C)
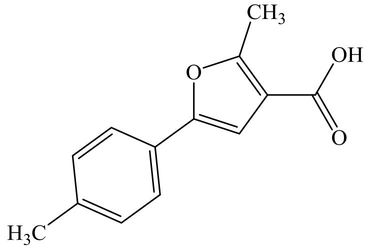	6.9	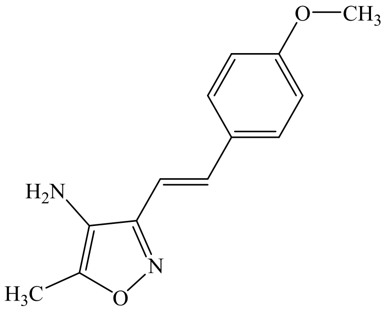	3.2
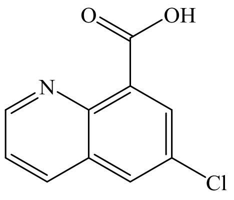	5.4	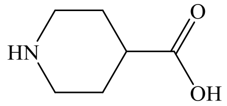	7.1
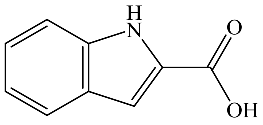	5.7	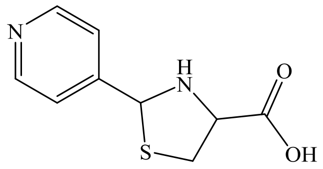	3.4
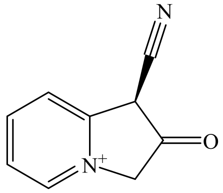	4.1	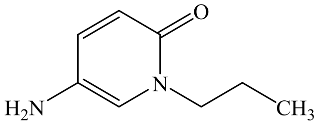	3.4
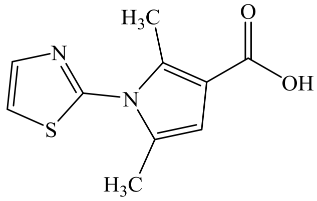	3.1	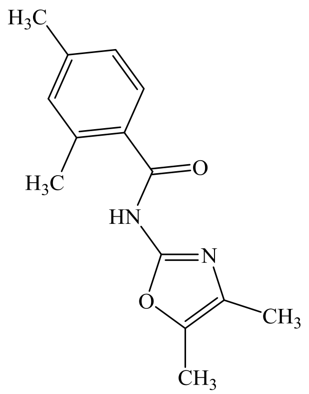	3.1
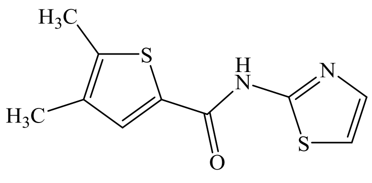	3.4		

Compound synthesis

Table S2. Indole diketo acid synthesis and starting material.

No	Chemical structure	No	Starting chemical structure
**19**	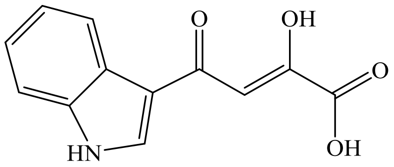	**19a**	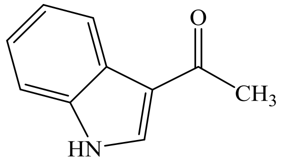
**20**	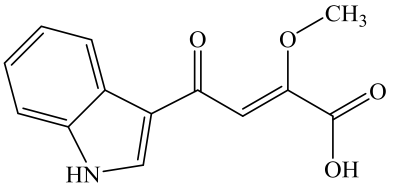		
**21**	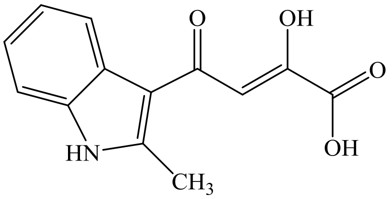	**21a**	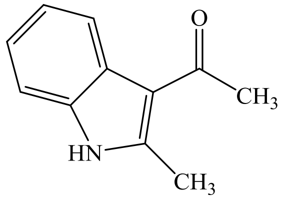
**22**	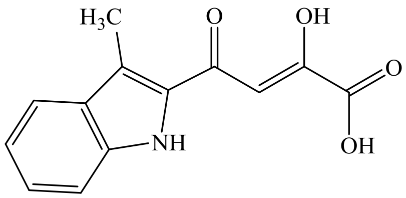	**22a**	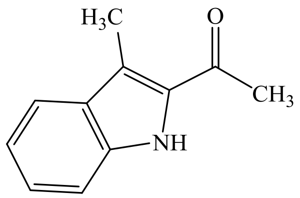
**23**	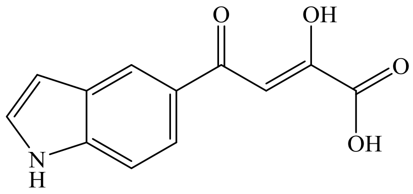	**23a**	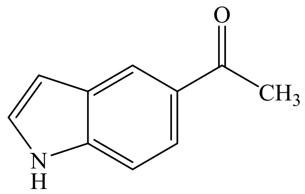

Table S2. Continued.

No	Chemical structure	No	Starting chemical structure
**24**	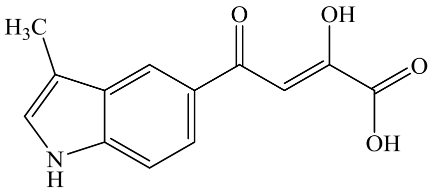	**24a**	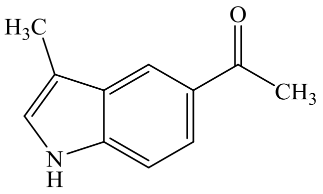
**25**	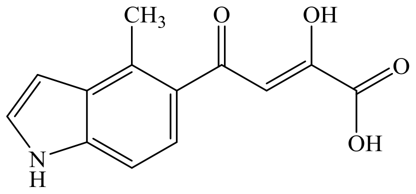	**25a**	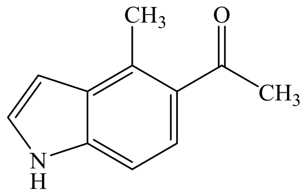
**26**	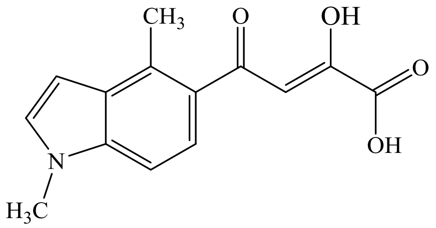	**26a**	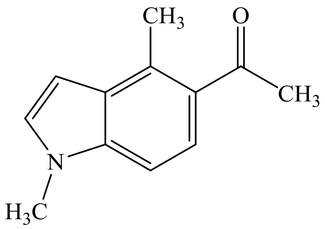
**27**	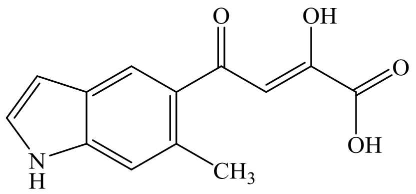	**27a**	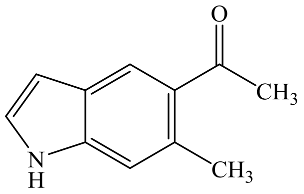

Scheme S1. General synthesis steps for indole diketo acids, **19-27**.

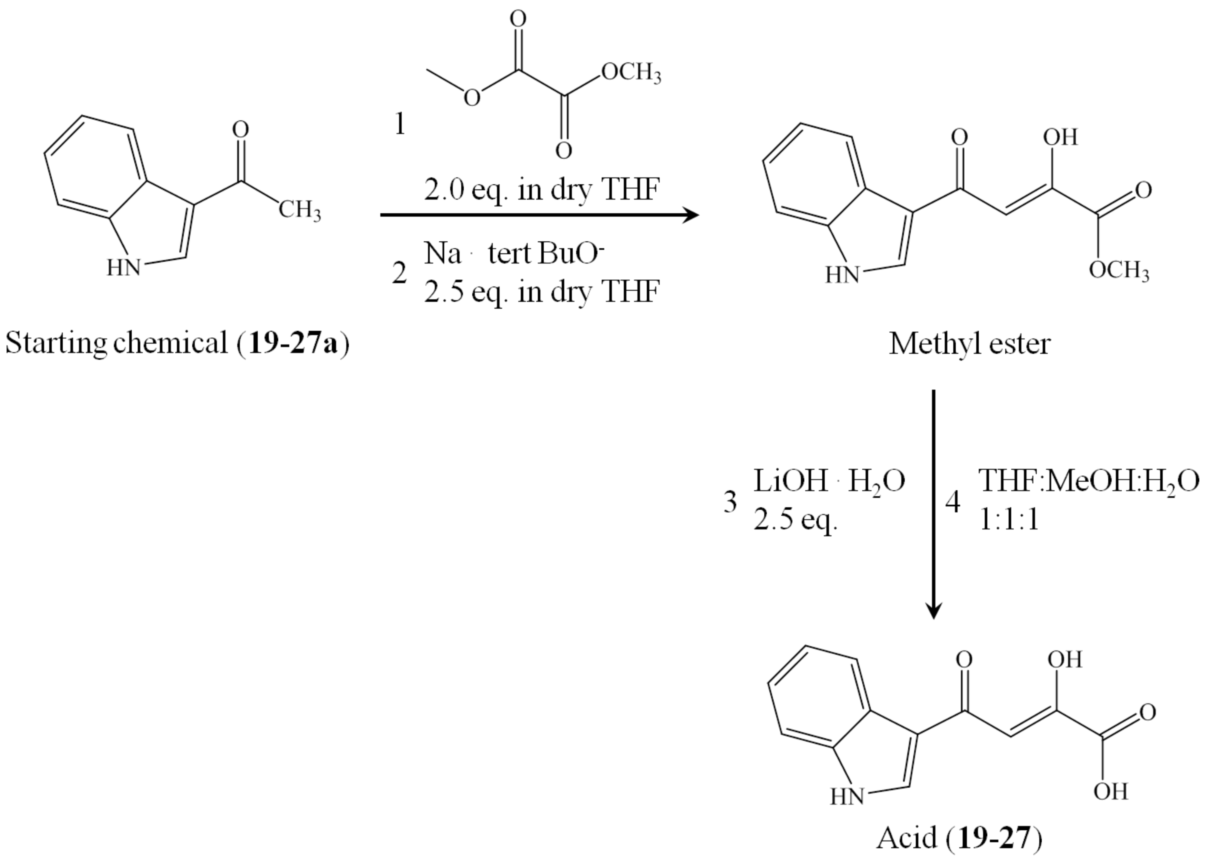

Compounds **19-27** were synthesized via the synthetic procedure shown in **Scheme 1** and described as follows: To a slurry of ^{t}BuONa (1.00 g, 5.36 mmol) in dry THF under N_2 (8 mL) a solution of starting material aldehyde (**19a-27a**, **Table S1**) in 2.0 mL anhydrous THF was added and via syringe. The mixture was stirred at room temperature for 30 min. and then dimethyl oxalate (2.2 equiv, 11.8 mmol, 1.39 g) was added. After 48 hours, the reaction mixture was quenched with 2.0 mL of 2 M HCl (aq) and extracted with EtOAc. The combined organic layer was washed with 10 mL brine solution, dried over anhydrous Na_2SO_4, filtered, and concentrated in vacuo. The crude mixture was purified by a silica gel column chromatography to afford the desired methyl-ester as an amorphous solid.

Methyl-ester of **19-27** was dissolved in 4 mL of 2:1:1 mixture of ($THF/CH_3OH/H_2O$) and xx mg (0.212 mmol) of LiOH monohydrate was added. The yellow solution was stirred for 6 h, and after

completion of hydrolysis the reaction mixture was acidified to pH 4 with 2 M HCl (aq), and the resulting mixture was extracted with 2x5 mL EtOAc. The combined organic phase was dried (Na_2SO_4), filtered, and concentrated to afford the desired acid product (**19-27**) as a solid.

Chemical information:

Compound **19**, (Z)-2-hydroxy-4-(1H-indol-3-yl)-4-oxobut-2-enoic acid: ^{1}H NMR (400 MHz, DMSO-*d6*) δ 8.29 (s, 1H), 8.18 (d, *J* = 7.2 Hz, 1H) , 7.47 (d, *J* = 7.5 Hz, 1H) , 7.23-7.16 (m, 2H), 2.50 (s, 3H); ^{13}C NMR (100 MHz, $CDCl_3$) δ 137.11, 134.74 (2C), 125.75, 123.14 (2C), 122.06 (2C), 121.76 (2C), 117.26, 112.50 (2C); MS (ESI) *m/z* 511.37 $(M + Na)^+$.

Compound **20**, (Z)-4-(1H-indol-3-yl)-2-methoxy-4-oxobut-2-enoic acid: ^{1}H NMR (400 MHz, DMSO-*d6*) δ 8.71 (s, 1H), 8.24 (d, *J* = 7.3 Hz, 1H) , 7.60-7.56 (m, 1H) , 7.36-7.24 (m, 3H), 6.94 (s, 1H), 3.89 (s, 3H); ^{13}C NMR (100 MHz, $CDCl_3$) δ 164.25, 163.70, 139.47, 138.25, 126.06, 123.94, 123.25, 122.09, 114.10, 111.59, 100.64, 33.90; MS (ESI) *m/z* 511.37 $(M + Na)^+$.

Compound **21**, (Z)-2-hydroxy-4-(2-methyl-1H-indol-3-yl)-4-oxobut-2-enoic acid: ^{1}H NMR (400 MHz, DMSO-*d6*) δ 8.05-7.95 (m, 1H), 7.49-7.38 (m, 1H) , 7.28-7.17 (m, 2H) , 6.86 (s, 1H), 2.74 (s, 3H); ^{13}C NMR (100 MHz, $CDCl_3$) δ 188.27, 167.92, 164.31, 146.84, 135.76, 126.74, 123.07, 122.60, 12081, 112.28, 110.70, 100.35, 15.73; MS (ESI) *m/z* 511.37 $(M + Na)^+$.

Compound **22**, (*Z*)-2-hydroxy-4-(3-methyl-1H-indol-2-yl)-4-oxobut-2-enoic acid: ^{1}H NMR (400 MHz, DMSO-*d6*) δ 8.05-7.95 (m, 1H), 7.49-7.38 (m, 1H) , 7.28-7.17 (m, 2H) , 6.86 (s, 1H), 2.74 (s, 3H); ^{13}C NMR (100 MHz, $CDCl_3$) δ 188.27, 167.92, 164.31, 146.84, 135.76, 126.74, 123.07, 122.60, 12081, 112.28, 110.70, 100.35, 15.73; MS (ESI) *m/z* 511.37 $(M + Na)^+$.

Compound **23**, (*Z*)-2-hydroxy-4-(1H-indol-5-yl)-4-oxobut-2-enoic acid: ^{1}H NMR (400 MHz, DMSO-*d6*) δ 8.44 (s, 1H), 7.83 (dd, *J* = 8.6, 1.2 Hz, 1H) , 7.56-7.45 (m, 2H) , 7.17 (s, 1H), 6.65-6.59 (m, 1H); ^{13}C NMR (100 MHz, $CDCl_3$) δ 163.94, 139.69, 128.13, 126.46, 122.88, 121.27, 112.45, 103.77, 98.27; MS (ESI) *m/z* 511.37 $(M + Na)^+$.

Compound **24**, (*Z*)-4-(1,4-dimethyl-1H-indol-5-yl)-2-hydroxy-4-oxobut-2-enoic acid: ^{1}H NMR (400 MHz, DMSO-*d6*) δ 7.59 (d, *J* = 8.7 Hz, 1H), 7.47-7.35 (m, 2H) , 6.87 (s, 1H), 6.69 (d, *J* = 2.9 Hz,

1H), 3.82 (s, 3H), 2.73 (s, 3H); ^{13}C NMR (100 MHz, $CDCl_3$) δ 183.43, 163.93, 138.22, 132.86, 131.44, 129.81, 126.36, 123.30, 108.22, 102.18, 101.67, 33.17, 17.62; MS (ESI) *m/z* 511.37 $(M + Na)^+$.

Compound **25**, (*Z*)-2-hydroxy-4-(3-methyl-1H-indol-5-yl)-4-oxobut-2-enoic acid: ^{1}H NMR (400 MHz, DMSO-*d6*) δ 8.33 (d, *J* = 8.2 Hz, 1H), 7.83 (d, *J* = 8.2 Hz, 1H) , 7.62-7.57 (m, 1H), 7.43-7.39 (m, 1H), 6.03 (s, 1H), 2.61 (s, 3H); ^{13}C NMR (100 MHz, $CDCl_3$) δ 183.43, 163.93, 138.22, 132.86, 131.44, 129.81, 126.36, 123.30, 108.22, 102.18, 101.67, 33.17, 17.62; MS (ESI) *m/z* 511.37 $(M + Na)^+$.

Compound **26**, (*Z*)-2-hydroxy-4-(6-methyl-1H-indol-5-yl)-4-oxobut-2-enoic acid: ^{1}H NMR (400 MHz, DMSO-*d6*) δ 11.34 (s, 1H), 8.14 (s, 1H), 7.45-7.38 (m, 1H) , 7.36-7.29 (m, 1H) , 6.95 (s, 1H), 6.56 (s, 1H), 2.61 (s, 3H); ^{13}C NMR (100 MHz, $CDCl_3$) δ 163.96, 138.79, 131.59, 127.74, 127.29, 126.08, 124.15, 114.58, 103.10, 101.77, 22.45; MS (ESI) *m/z* 511.37 $(M + Na)^+$.

Compound **27**, (*Z*)-2-hydroxy-4-(4-methyl-1H-indol-5-yl)-4-oxobut-2-enoic acid: ^{1}H NMR (400 MHz, DMSO-*d6*) δ 7.55 (d, *J* = 8.5 Hz, 1H), 7.50-7.44 (m, 1H) , 7.37 (d, *J* = 8.5 Hz, 1H), 6.89 (s, 1H), 6.75-6.67 (m, 1H), 2.75 (s, 3H); ^{13}C NMR (100 MHz, $CDCl_3$) δ 183.43, 163.93, 138.22, 132.86, 131.44, 129.81, 126.36, 123.30, 108.22, 102.18, 101.67, 33.17, 17.62; MS (ESI) *m/z* 511.37 $(M + Na)^+$.

Table S3. Crystal data collection and refinement statistics.

Statistics	GlcB C619A-**1** complex	GlcB C619A-**2** complex	GlcB C619A-**3** complex	GlcB C619A-**4** complex
PDB ID	5C9R	5T8G	5C9U	5C9W
Data Collection				
Wavelegnth (Å)	0.97918	0.97915	0.97947	0.97946
Space group	$P4_32_12$	$P4_32_12$	$P4_32_12$	$P4_32_12$
Cell dimensions				
a, b, c (Å)	78.50, 78.50, 224.28	78.85, 78.85, 224.93	78.74, 78.74, 224.66	78.21, 78.21, 223.82
α, β, γ (°)	90, 90, 90	90, 90, 90	90, 90, 90	90, 90, 90
Unique reflections	47444	46135	49254	40353
R_{sym} or R_{merge}	0.189 (0.799)	0.160 (0.574)	0.114 (0.530)	0.203 (0.724)
$I/\sigma I$	7.63 (1.01)	23.3 (4.07)	24.21(6.95)	18.82 (3.66)
Completeness (%)	98.5 (96.9)	99.4 (98.4)	93.48 (97.19)	96.03 (98.92)
Redudancy	6.5 (4.1)	13.5 (11.4)	4.5 (4.3)	9.4 (8.3)
Refinement				
Resolution (Å)	44.57-2.00 (2.04-2.00)	45.78-2.04 (2.08-2.04)	44.68-1.95 (1.99-1.95)	44.43-2.09 (2.14-2.09)
R_{work}/R_{free}	0.187/0.250	0.162/0.208	0.183/0.236	0.176/0.235
No. of atoms				
Protein	5480	5475	5466	5457
Ligand/ion	14	18	17	16
Water	639	580	620	593
B factors				
Protein	31.3	29.1	35.5	29.3
Mg	19.4	28.6	34.9	25.7
Small molecule	31.1	25.7	39.2	34.2
Solvent	36.7	39.2	42.9	35.2
Rmsd				
Bond length (Å)	0.008	0.008	0.009	0.009
Bond angles (°)	1.15	1.11	1.19	1.06
Ramachandran				
Favored (%)	96.9	97.3	96.5	97.4
Allowed	2.4	2.4	2.6	2.3
Outliers (%)	0.7	0.3	1.0	0.3
Clashscore	3.94	2.93	2.11	2.67

Statistics for the highest-resolution shell are shown in parentheses.

Table S3. Continued.

Statistics	GlcB C619A-**5** complex	GlcB C619A-**6** complex	GlcB C619A-**7** complex	GlcB C619A-**8** complex
PDB ID	5C9X	5CAH	5E9X	5C7V
Data Collection				
Wavelength (Å)	0.97946	1.0000	1.0076	0.91943
Space group	$P4_32_12$	$P4_32_12$	$P4_32_12$	$P4_32_12$
Cell dimensions				
a, *b*, *c* (Å)	79.36, 79.36, 225.31	80.69,80.69, 226.29	81.66, 81.66, 226.56	78.88, 78.88, 224.66
α, β, γ (°)	90, 90, 90	90, 90, 90	90, 90, 90	90, 90, 90
Unique reflections	40993	33871	56725	25218
R_{sym} or R_{merge}	0.120 (0.597)	0.103 (0.598)	0.367 (0.651)	0.205 (0.738)
$I/\sigma I$	26.24 (4.42)	15.49 (2.89)	14.61 (2.28)	18.15 (5.60)
Completeness (%)	95.33 (94.74)	99.45 (99.16)	98.63 (92.87)	99.37 (98.87)
Redundancy	9.4 (8.3)	6.6 (6.6)	8.6 (4.9)	13.5 (13.6)
Refinement				
Resolution (Å)	35.14-2.10 (2.15-2.10)	39.47-2.30 (2.34-2.30)	35.87-1.94 (2.01-1.94)	49.96-2.50 (2.60-2.50)
R_{work}/R_{free}	0.173/0.237	0.202/0.290	0.219/0.284	0.157/0.244
No. of atoms				
Protein	5489	5404	5488	5475
Ligand/ion	15	16	15	11
Water	597	498	483	475
B factors				
Protein	40.2	41.5	51.3	34.6
Mg	33.8	38.1	54.3	32.2
Small molecule	37.5	36.6	46.3	30.9
Solvent	46.0	43.9	54.6	37.0
Rmsd				
Bond length (Å)	0.007	0.008	0.007	0.007
Bond angles (°)	1.13	1.08	1.06	1.19
Ramachandran				
Favored (%)	95.9	95.4	96.0	95.1
Allowed	3.5	4.3	3.1	3.5
Outliers (%)	0.6	0.3	0.9	1.4
Clashscore	3.38	3.10	3.84	4.68

Statistics for the highest-resolution shell are shown in parentheses.

Table S3. Continued.

Statistics	GlcB C619A-**9** complex	GlcB C619A-**10** complex	GlcB C619A-**11** complex	GlcB C619A-**12** complex
PDB ID	5CAK	5CJN	5CC3	5CBB
Data Collection				
Wavelength (Å)	1.0000	0.97949	1.0332	0.97944
Space group	$P4_32_12$	$P4_32_12$	$P4_32_12$	$P4_32_12$
Cell dimensions				
a, *b*, *c* (Å)	78.24, 78.24, 223.57	78.81, 78.81, 223.82	78.24, 78.24, 224.15	80.82, 80.82, 225.99
α, β, γ (°)	90, 90, 90	90, 90, 90	90, 90, 90	90, 90, 90
Unique reflections	47969	36629	31589	50605
R_{sym} or R_{merge}	0.146 (0.539)	0.111 (0.423)	0.153 (0.183)	0.164 (0.724)
$I/\sigma I$	23.12 (2.83)	25.49 (1.70)	27.54 (3.30)	23.45 (3.03)
Completeness (%)	99.04 (94.39)	97.62 (94.86)	87.26 (67.06)	99.81 (98.89)
Redundancy	6.6 (6.6)	8.1 (7.1)	6.3 (2.7)	13.1 (9.4)
Refinement				
Resolution (Å)	49.58-1.99 (2.00-1.99)	44.65-2.19 (2.24-2.19)	44.46-2.20 (2.27-2.20)	45.53-2.01 (2.05-2.01)
R_{work}/R_{free}	0.164/0.221	0.171/0.228	0.177/0.249	0.214/0.275
No. of atoms				
Protein	5494	5447	5473	5454
Ligand/ion	16	24	15	18
Water	695	548	580	522
B factors				
Protein	17.7	25.2	30.5	30.4
Mg	11.9	33.7	20.5	24.0
Small molecule	22.4	23.5	33.4	26.8
Solvent	26.1	32.4	36.2	33.2
Rmsd				
Bond length (Å)	0.007	0.008	0.008	0.007
Bond angles (°)	1.08	1.10	1.16	1.03
Ramachandran				
Favored (%)	96.8	96.6	96.2	95.8
Allowed	2.8	2.7	2.8	3.4
Outliers (%)	0.4	0.7	1.0	0.9
Clashscore	3.10	3.68	5.13	3.96

Statistics for the highest-resolution shell are shown in parentheses.

Table S3 Continued.

Statistics	GlcB C619A-**13** complex	GlcB C619A-**14** complex	GlcB C619A-**15** complex	GlcB C619A-**16** complex
PDB ID	5CJM	5CBI	5CBJ	5CCZ
Data Collection				
Wavelength (Å)	0.97960	0.97924	0.97947	0.97944
Space group	$P4_32_12$	$P4_32_12$	$P4_32_12$	$P4_32_12$
Cell dimensions				
a, b, c (Å)	80.76, 80.76, 226.6	79.8, 79.8, 225.06	79.03,79.03, 225.86	81.51, 81.51, 227.03
α, β, γ (°)	90, 90, 90	90, 90, 90	90, 90, 90	90, 90, 90
Unique reflections	42208	50244	46965	42379
R_{sym} or R_{merge}	0.140 (0.440)	0.165 (0.756)	0.236 (0.791)	0.151 (0.732)
$I/\sigma I$	13.38 (1.18)	25.86 (1.49)	15.81 (2.31)	30.62 (3.46)
Completeness (%)	98.19 (86.95)	98.38 (88.26)	89.10 (90.15)	97.48 (93.87)
Redundancy	16.5 (14.6)	8.7 (4.7)	5.4 (3.3)	15.9 (11.6)
Refinement				
Resolution (Å)	40.22-2.13 (2.18-2.13)	45.11-1.99 (2.03-1.99)	44.87-1.96 (2.00-1.96)	35.88-2.14 (2.19-2.14)
R_{work}/R_{free}	0.208/0.299	0.193/0.241	0.208/0.272	0.193/0.250
No. of atoms				
Protein	5446	5503	5421	5451
Ligand/ion	13	41	13	15
Water	376	451	535	489
B factors				
Protein	29.2	36.3	35.9	30.9
Mg	20.5	23.5	26.6	24.9
Small molecule	29.1	38.9	46.4	30.2
Solvent	28.2	40.9	42.4	34.6
Rmsd				
Bond length (Å)	0.008	0.009	0.007	0.008
Bond angles (°)	1.13	1.75	1.12	1.13
Ramachandran				
Favored (%)	96.2	96.5	97.2	96.2
Allowed	3.3	2.8	2.0	3.3
Outliers (%)	0.6	0.7	0.9	0.6
Clashscore	6.82	3.55	4.36	3.87

Statistics for the highest-resolution shell are shown in parentheses.

Table S3 Continued.

Statistics	GlcB C619A-**17** complex	GlcB C619A-**18** complex	GlcB C619A-**19** complex	GlcB C619A-**23** complex
PDB ID	5DX7	5CW	5DRC	5DRI
Data Collection				
Wavelength (Å)	0.97946	0.97947	1.0332	1.5
Space group	$P4_32_12$	$P4_32_12$	$P4_32_12$	$P4_32_12$
Cell dimensions				
a, *b*, *c* (Å)	78.95, 78.95, 225.13	78.38, 78.38, 225.19	78.96, 78.96, 224.97	79.30,79.30, 224.98
α, β, γ (°)	90, 90, 90	90, 90, 90	90, 90, 90	90, 90, 90
Unique reflections	38777	40981	33398	17322
R_{sym} or R_{merge}	0.129 (0.627)	0.209 (0.606)	0.108 (0.439)	0.226 (0.836)
$I/\sigma I$	19.64 (3.42)	17.58 (4.93)	21.43 (6.64)	20.02 (4.36)
Completeness (%)	90.83 (96.12)	88.45 (98.43)	87.82 (92.20)	93.53 (98.44)
Redundancy	8.4 (8.5)	4.8 (5.7)	6.6 (7.5)	12.9 (14.0)
Refinement				
Resolution (Å)	34.94-2.10 (2.15-2.10)	44.59-2.03 (2.08-2.03)	44.78-2.18 (2.25-2.18)	44.91-2.80 (2.98-2.80)
R_{work}/R_{free}	0.184/0.256	0.205/0.292	0.172/0.250	0.171/0.296
No. of atoms				
Protein	5460	5448	5473	5419
Ligand/ion	15	15	38	18
Water	482	625	511	425
B factors				
Protein	33.4	29.5	23.4	30.0
Mg	26.1	26.7	21.2	19.0
Small molecule	49.8	36.8	23.9	20.9
Solvent	37.9	38.2	29.0	29.7
Rmsd				
Bond length (Å)	0.008	0.010	0.007	0.011
Bond angles (°)	1.15	1.16	1.12	1.11
Ramachandran				
Favored (%)	96.6	96.7	96.3	93.8
Allowed	3.0	2.4	2.7	4.3
Outliers (%)	0.4	0.9	1.0	1.9
Clashscore	2.94	5.43	3.93	6.57

Statistics for the highest-resolution shell are shown in parentheses.

Table S3. Continued.

Statistics	GlcB C619A-**25** complex	GlcB WT apo	GlcB WT-malate complex	GlcB G459A C619A-malate complex
PDB ID	5ECV	5H8P	5H8U	5H8M
Data Collection				
Wavelength (Å)	0.987	0.9795	0.97880	0.97934
Space group	$P4_32_12$	$P4_32_12$	$P4_12_12$	$P4_32_12$
Cell dimensions				
a, *b*, *c* (Å)	77.79, 77.79, 222.98	81.79, 81.79, 227.08	120.66, 120.66, 232.22	80.26, 80.26, 226.44
α, β, γ (°)	90, 90, 90	90, 90, 90	90, 90, 90	90, 90, 90
Unique reflections	40884	40314	66430	34332
R_{sym} or R_{merge}	0.128 (0.681)	0.119 (0.972)	0.108 (0.94)	0.418 (0.935)
$I/\sigma I$	11.6 (1.67)	11.66 (3.44)	14.96 (0.89)	16.75 (1.13)
Completeness (%)	97.57 (98.43)	88.21 (83.42)	85.51 (11.96)	89.53 (45.14)
Redundancy	6.5 (4.3)	12.6 (13.1)	14.4 (16.3)	19.4 (17.9)
Refinement				
Resolution (Å)	44.21-2.08 (2.13-2.08)	45.96-2.10 (2.15-2.10)	37.36-2.85 (2.92-2.85)	45.36-2.70 (2.82-2.70)
R_{work}/R_{free}	0.178/0.239	0.251/0.312	0.226/0.276	0.226/0.322
No. of atoms				
Protein	5456	5387	10988	5421
Ligand/ion	19		17	10
Water	522	102	60	58
B factors				
Protein	28.9	34.6	78.9	47.8
Mg	17.5		61.3	30.1
Small molecule	21.8		66.0	43.3
Solvent	36.1	27.7	64.8	37.6
Rmsd				
Bond length (Å)	0.009	0.008	0.003	0.008
Bond angles (°)	1.12	1.16	0.62	1.14
Ramachandran				
Favored (%)	96.8	94.7	94.7	92.6
Allowed	2.6	4.2	4.2	6.2
Outliers (%)	0.7	1.0	1.0	1.2
Clashscore	4.60	7.07	5.94	8.07

Statistics for the highest-resolution shell are shown in parentheses.

Figure S1. Crystal structure of GlcB complexed with Group 1 fragments showing the electron density of omit F_o-F_c map for the bound fragment contoured as following: (**A**) **1** at 2.5 σ, (**B**) **2** at 3.5 σ, (**C**) **3** at 2.5 σ, (**D**) **4** at 2.5 σ, and (**E**) **5** at 4 σ. Atom colors: chartreuse, magnesium; darkgrey/black, carbon; red, oxygen; blue, nitrogen; light green, fluorine; green, chlorine; brown, bromine; yellow, sulfur. Images are rendered in CHIMERA.

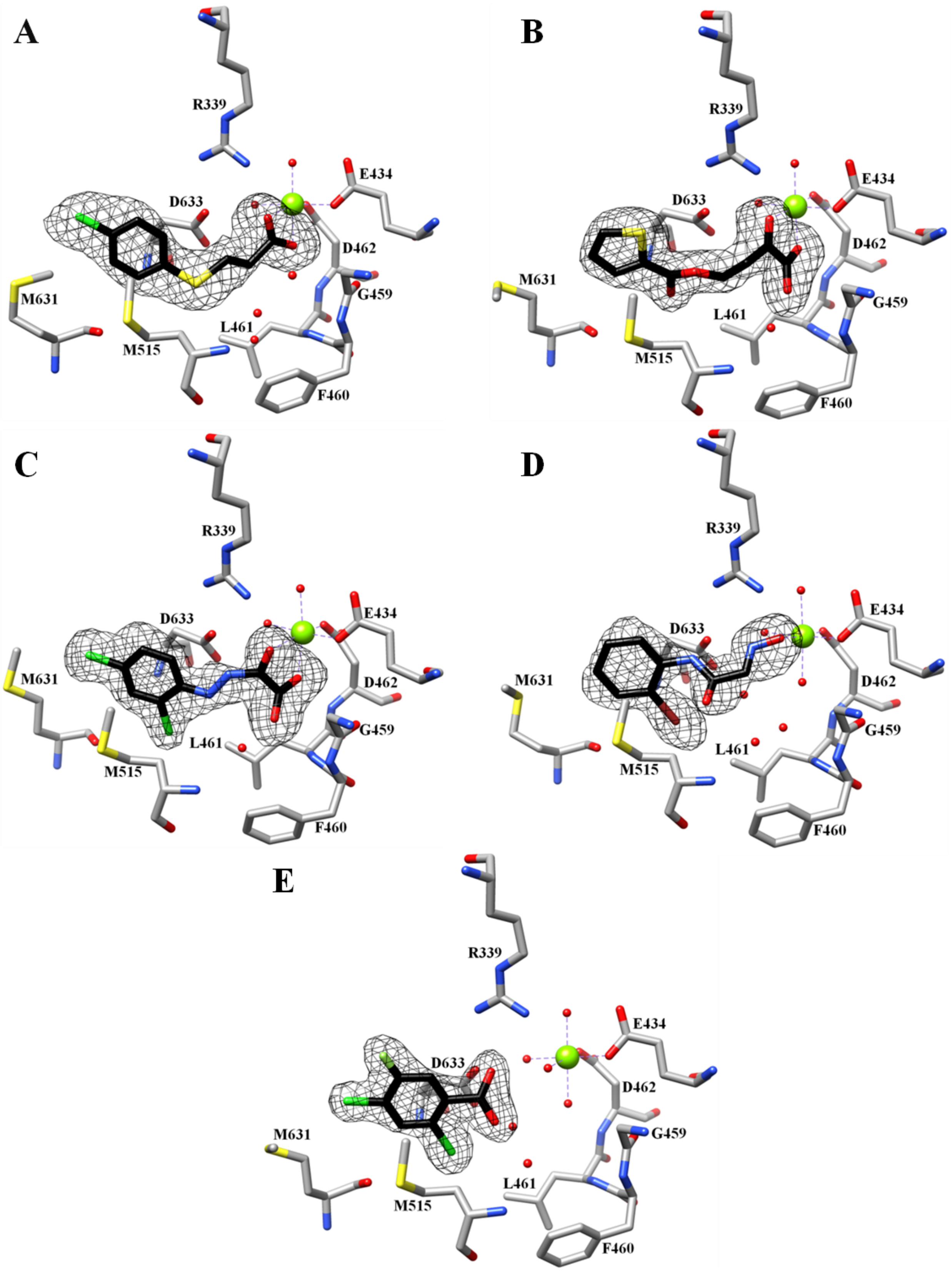
A
R339
E434
D633
D462
M631
G459
L461
M515
F460
B
R339
E434
D633
D462
M631
G459
L461
M515
F460
C
R339
E434
D633
M631
D462
G459
L461
M515
F460
D
R339
E434
D633
M631
D462
G459
L461
M515
F460
E
R339
E434
D633
D462
M631
G459
L461
M515
F460

Figure S2. Crystal structure of GlcB complexed with Group 2 fragments showing the electron density of omit F_o-F_c map for the bound fragment contoured as following: (A) **6** at 3.65 σ, (B) **7** at 4 σ, (C) **8** at 3 σ, (D) **9** at 2.8 σ, (E) **10** at 3.3 σ, (F) **11** at 2.5 σ, (G) **12** at 2.55 σ, (H) **13** at 2.55 σ, (I) **14** at 2.5 σ, (J) **15** at 2.7 σ, (K) **16** at 3 σ, (L) **17** at 2.5 σ, and (M) **18** at 2.5 σ. Atom colors: chartreuse, magnesium; sandy brown/sienna, carbon; red, oxygen; blue, nitrogen; light green, fluorine; green, chlorine; brown, bromine; yellow, sulfur. Images are rendered in CHIMERA.

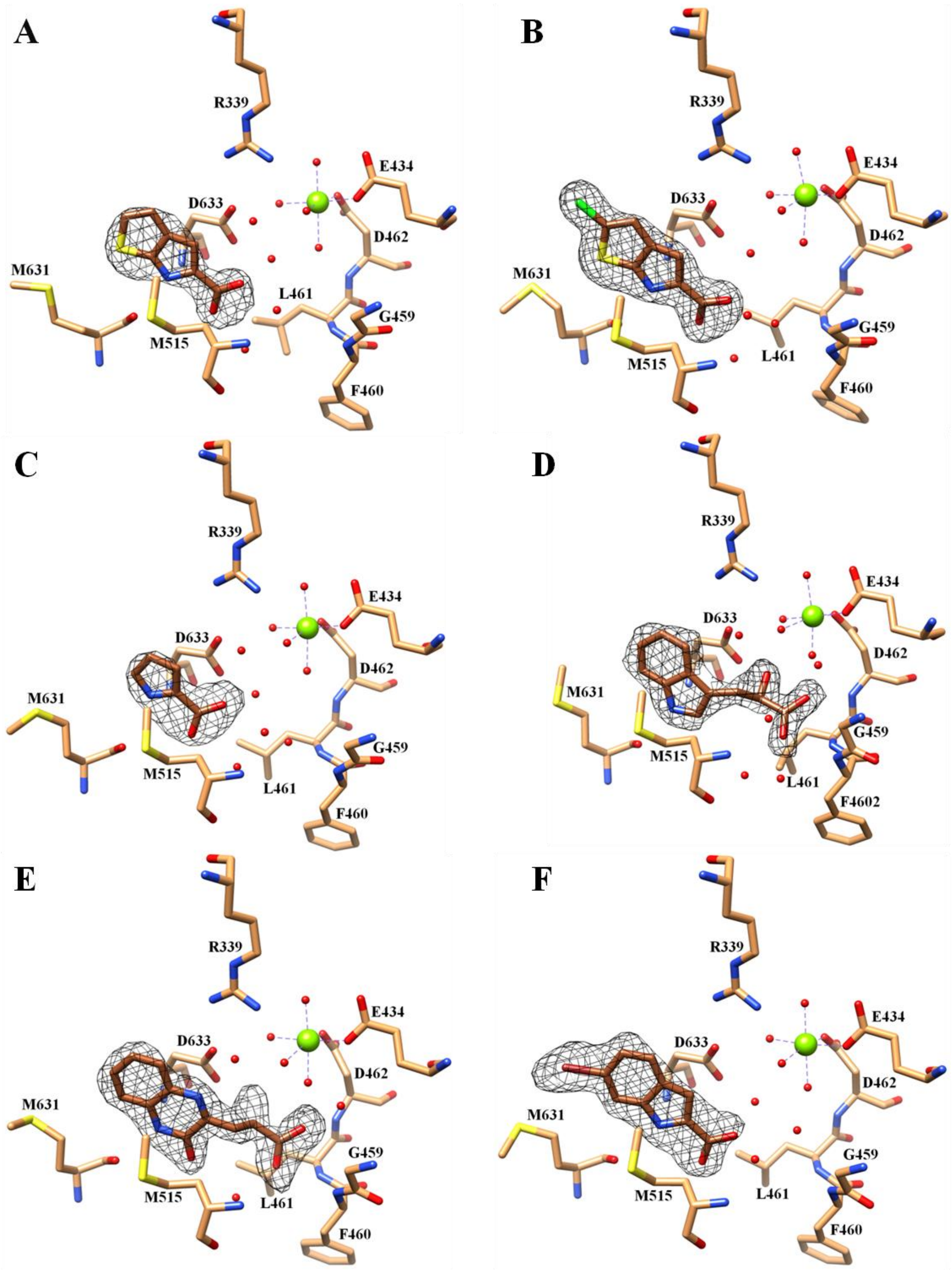
A
R339
E434
D633
D462
M631
L461
G459
M515
F460
B
R339
E434
D633
D462
M631
G459
L461
M515
F460
C
R339
E434
D633
D462
M631
G459
M515
L461
F460
D
R339
E434
D633
D462
M631
G459
M515
L461
F4602
E
R339
E434
D633
D462
M631
G459
M515
L461
F460
F
R339
E434
D633
D462
M631
G459
M515
L461
F460

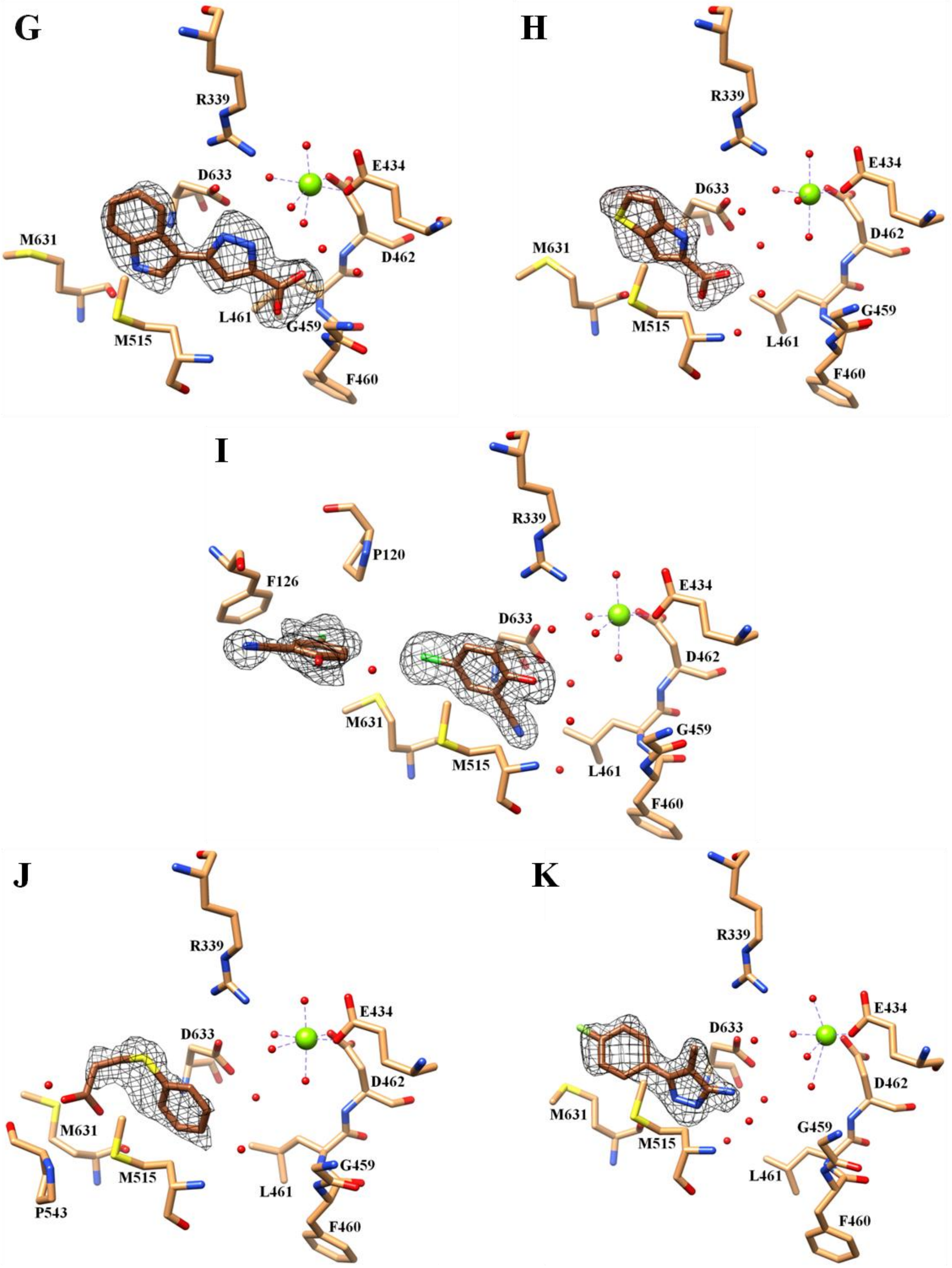
G
R339
D633
E434
M631
D462
L461
G459
M515
F460
H
R339
E434
D633
D462
M631
G459
M515
L461
F460
I
R339
P120
F126
E434
D633
D462
M631
G459
M515
L461
F460
J
R339
E434
D633
D462
M631
G459
M515
L461
P543
F460
K
R339
E434
D633
D462
M631
G459
M515
L461
F460

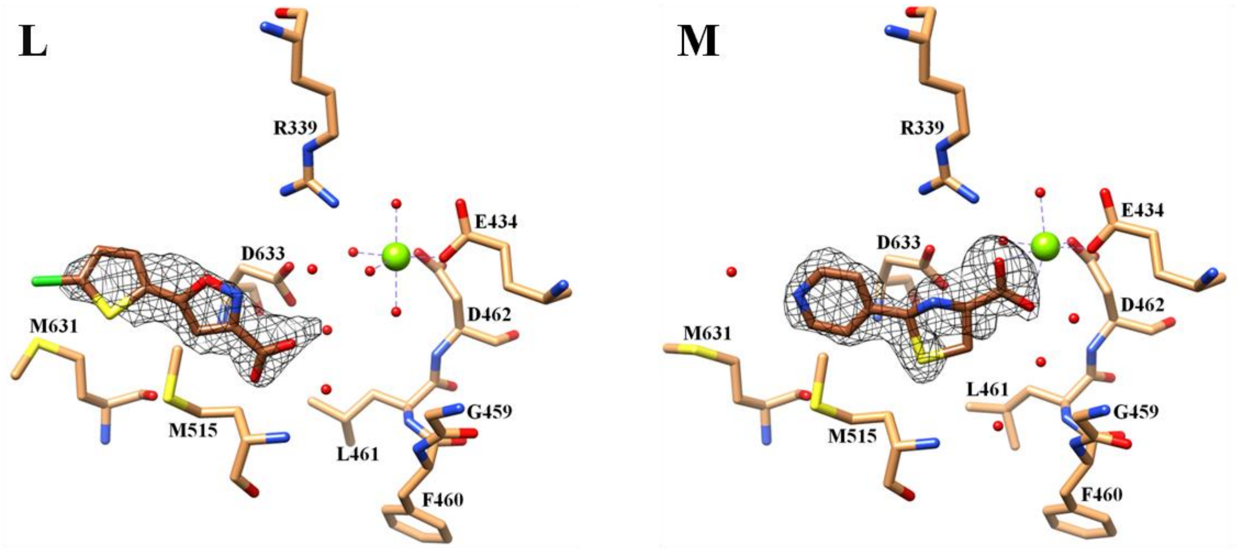
L
R339
E434
D633
D462
M631
M515
G459
L461
F460
M
R339
E434
D633
D462
M631
L461
G459
M515
F460

Figure S3. Crystal structure of GlcB complexed with Group 1 fragments showing the hydrogen bond and metal chelating interactions among the fragment, water molecules, and the active site residues: (**A**) **1**, (**B**) **2**, (**C**) **3**, (**D**) **4**, and (**E**) **5**. Atom colors: chartreuse, magnesium; dark grey/black, carbon; red, oxygen; blue, nitrogen; light green, fluorine; green, chlorine; brown, bromine; yellow, sulfur. Images are rendered in CHIMERA.

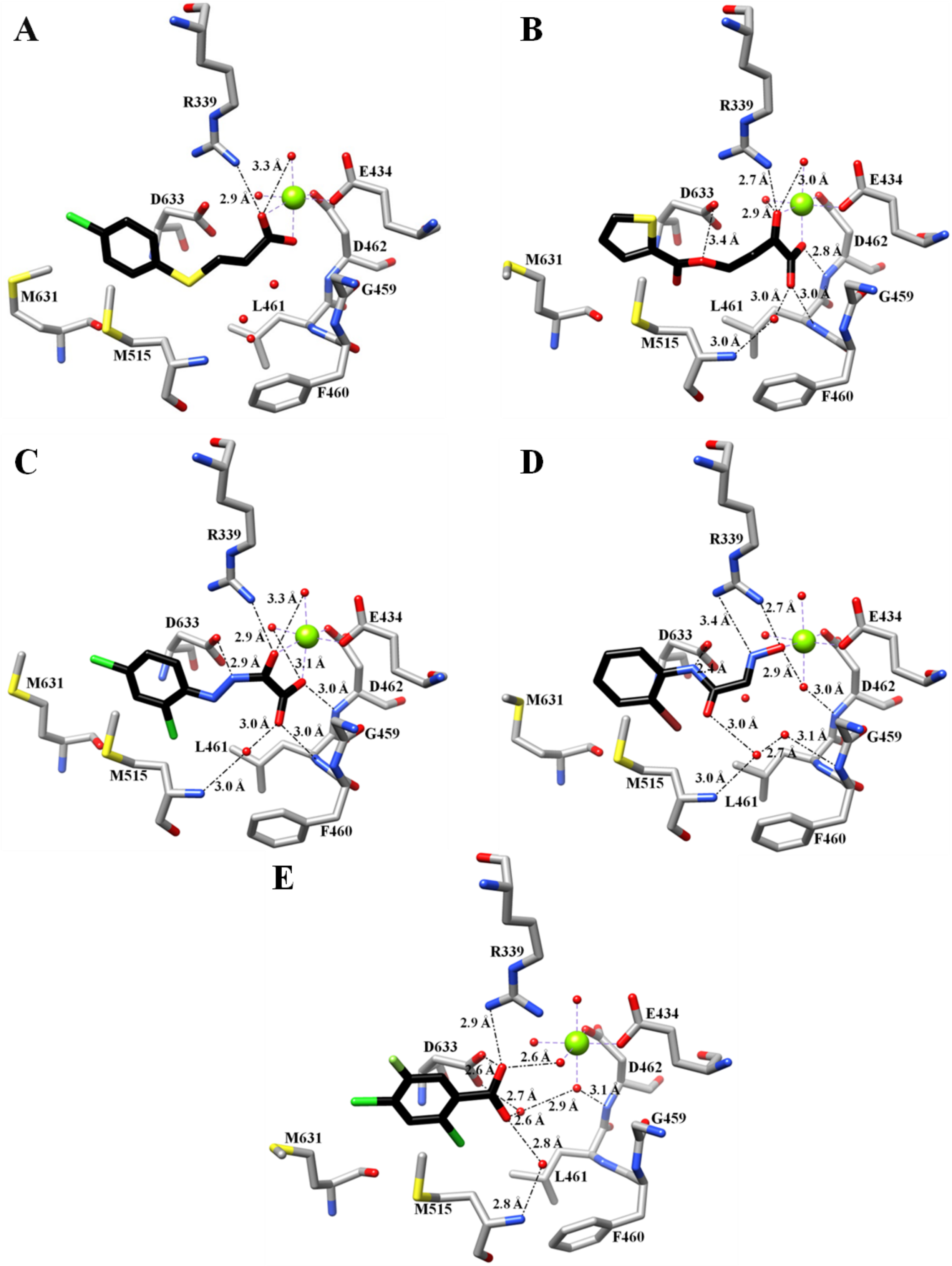
A
R339
3.3 Å
2.9 Å
E434
D633
D462
M631
G459
L461
M515
F460
B
R339
2.7 Å
3.0 Å
E434
D633
2.9 Å
3.4 Å
D462
M631
2.8 Å
3.0 Å
3.0 Å
G459
L461
3.0 Å
M515
F460
C
R339
3.3 Å
E434
D633
2.9 Å
2.9 Å
3.1 Å
M631
3.0 Å
D462
3.0 Å
3.0 Å
G459
L461
M515
3.0 Å
F460
D
R339
2.7 Å
3.4 Å
E434
D633
2.4 Å
2.9 Å
M631
3.0 Å
D462
3.0 Å
3.1 Å
G459
2.7 Å
3.0 Å
M515
L461
F460
E
R339
2.9 Å
E434
D633
2.6 Å
2.6 Å
D462
2.7 Å
3.1 Å
2.9 Å
2.6 Å
G459
M631
2.8 Å
L461
M515
2.8 Å
F460

Figure S4. Crystal structure of GlcB complexed with Group 2 fragments showing the H-bond and metal chelating interactions among the fragment, water molecules, and the active site residues: (A) **6**, (B) **7**, (C) **8**, (D) **9**, (E) **10**, (F) **11**, (G) **12**, (H) **13**, (I) **14**, (J) **15**, (K) **16**, (L) **17**, and (M) **18**. Atom colors: chartreuse, magnesium; sandy brown/sienna, carbon; red, oxygen; blue, nitrogen; light green, fluorine; green, chlorine; brown, bromine; yellow, sulfur. Images are rendered in CHIMERA.

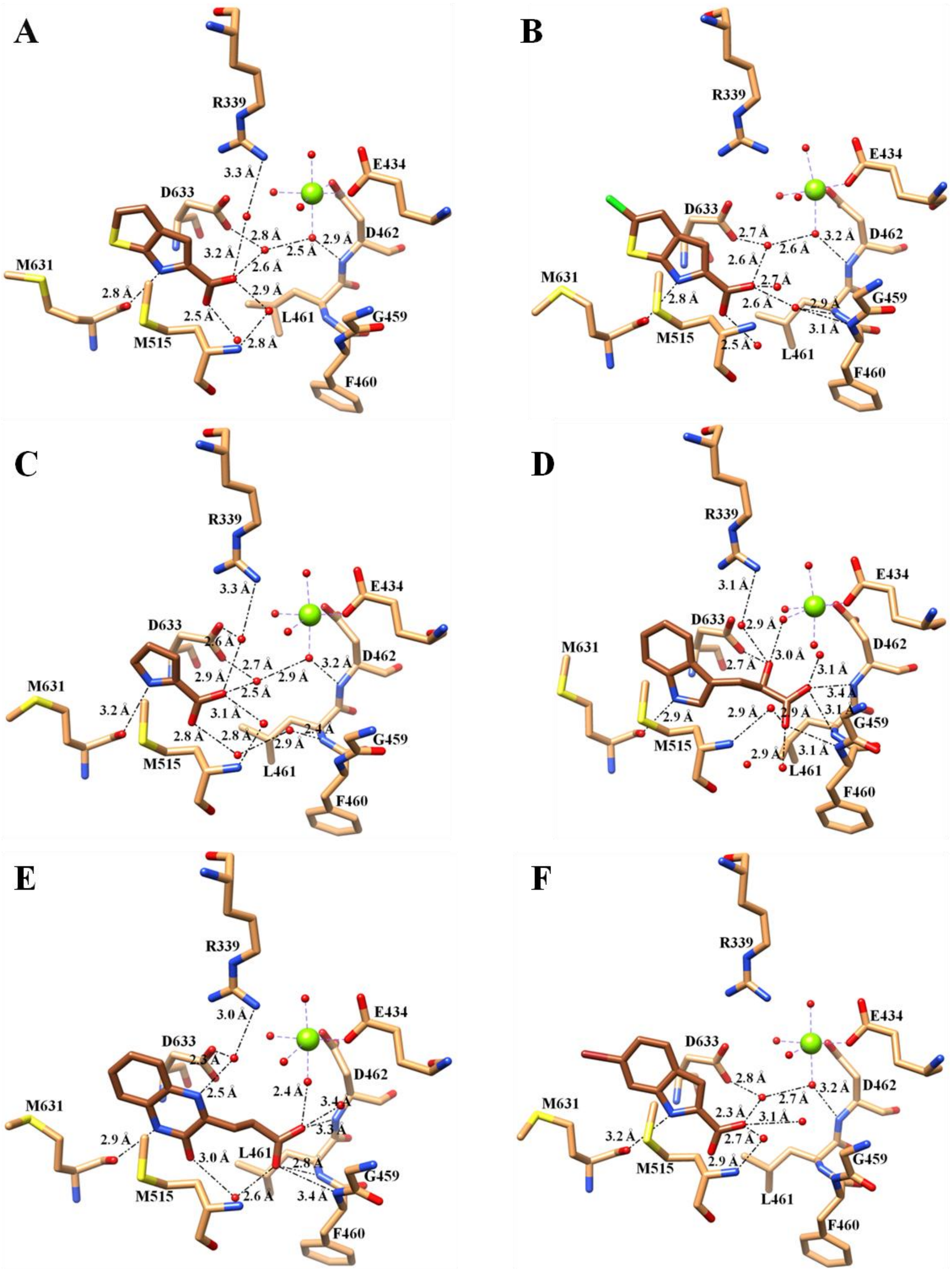
A
R339
D633
E434
D462
M631
M515
L461
G459
F460
B
R339
D633
E434
D462
M631
M515
L461
G459
F460
C
R339
D633
E434
D462
M631
M515
L461
G459
F460
D
R339
D633
E434
D462
M631
M515
L461
G459
F460
E
R339
D633
E434
D462
M631
M515
L461
G459
F460
F
R339
D633
E434
D462
M631
M515
L461
G459
F460

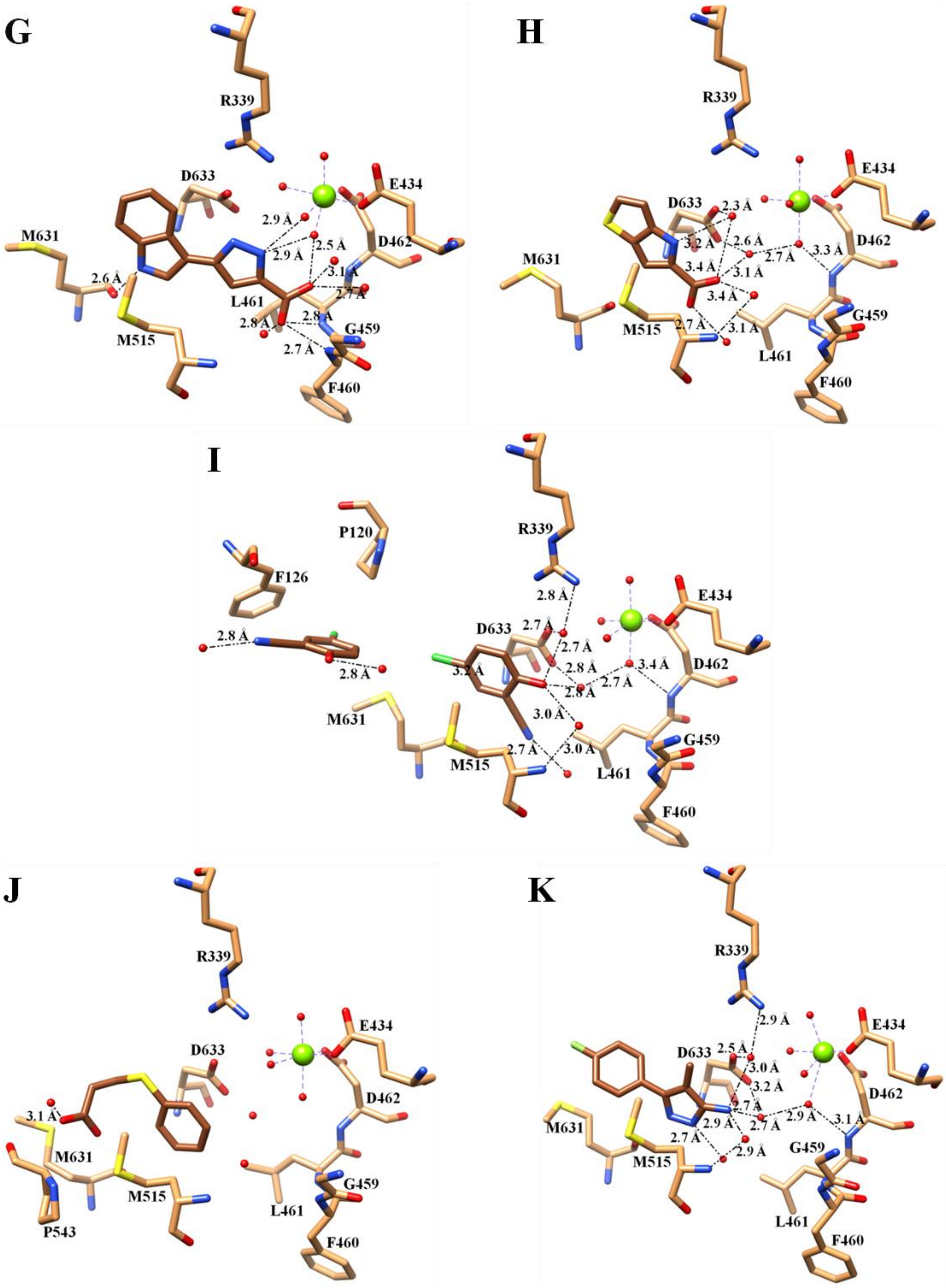
G
R339
D633
E434
M631
D462
L461
G459
M515
F460
H
R339
E434
D633
D462
M631
M515
G459
L461
F460
I
P120
F126
R339
E434
D633
D462
M631
G459
M515
L461
F460
J
R339
E434
D633
D462
M631
M515
G459
L461
P543
F460
K
R339
E434
D633
D462
M631
M515
G459
L461
F460

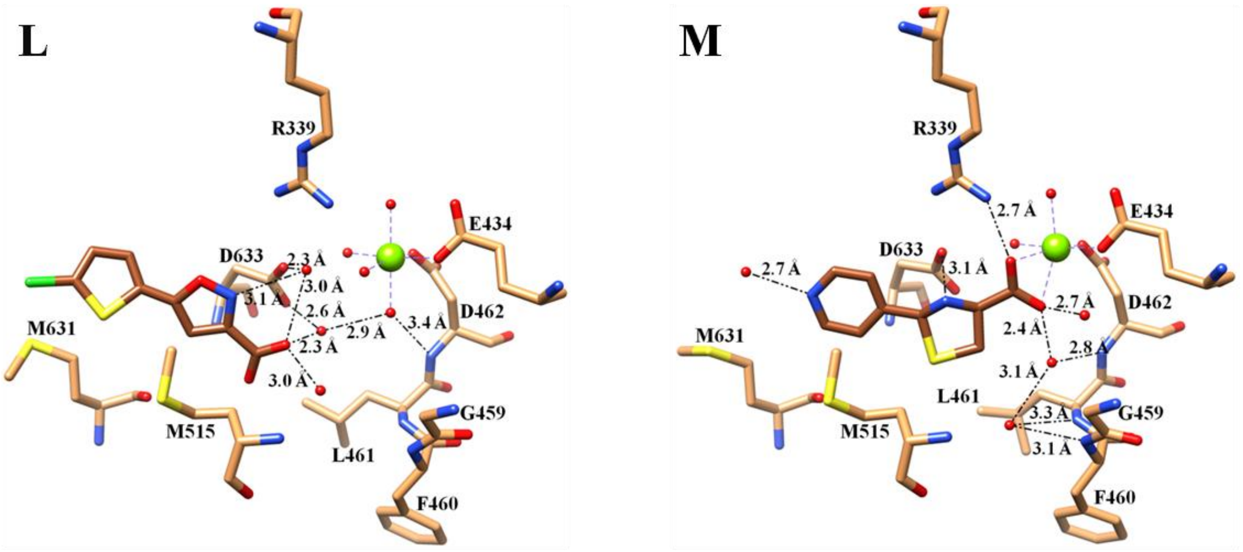
L
R339
E434
D633
2.3 Å
3.0 Å
3.1 Å
2.6 Å
2.9 Å
3.4 Å
D462
2.3 Å
M631
3.0 Å
G459
M515
L461
F460
M
R339
2.7 Å
E434
2.7 Å
D633
3.1 Å
2.7 Å
D462
2.4 Å
2.8 Å
M631
3.1 Å
L461
3.3 Å
G459
M515
3.1 Å
F460

Figure S5. Crystal structure of GlcB complexed with indole diketo acids showing the H-bond and metal chelating interactions among the indole diketo acid molecule, water molecules, and the active site residues: (A) **19**, (B) **23**, and (C) **25**. Atom colors: chartreuse, magnesium; light blue/sky blue, carbon; red, oxygen; blue, nitrogen; yellow, sulfur. Images are rendered in CHIMERA.

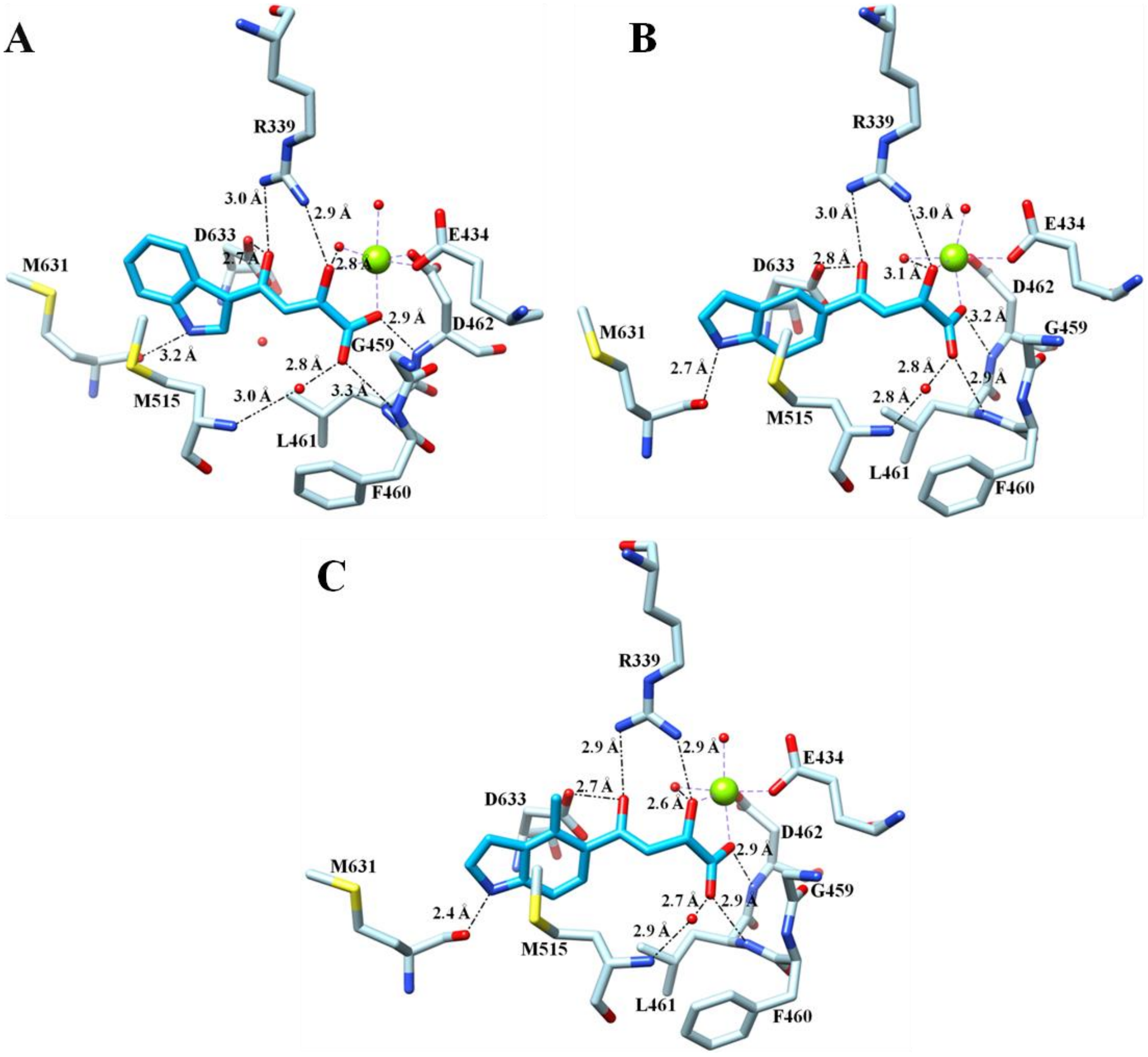

Supplement: Supplemental Data [file 10.1074_M116.750877_jbc.M116.750877-1.pdf]
